# Assessment of left atrial fibrosis progression in canines following rapid ventricular pacing using 3D late gadolinium enhanced CMR images

**DOI:** 10.1371/journal.pone.0269592

**Published:** 2022-07-08

**Authors:** Nadia A. Farrag, Rebecca E. Thornhill, Frank S. Prato, Allan C. Skanes, Rebecca Sullivan, David Sebben, John Butler, Jane Sykes, Benjamin Wilk, Eranga Ukwatta

**Affiliations:** 1 Department of Systems & Computer Engineering, Carleton University, Ottawa, ON, Canada; 2 Department of Radiology, University of Ottawa, Ottawa, ON, Canada; 3 Department of Medical Imaging and Medical Biophysics, University of Western Ontario, London, ON, Canada; 4 Lawson Health Research Institute, London, ON, Canada; 5 Department of Medicine, University of Western Ontario, London, ON, Canada; 6 School of Engineering, University of Guelph, Guelph, ON, Canada; Faculty of Medical Science - State University of Campinas, BRAZIL

## Abstract

**Background:**

Atrial fibrillation (AF) is associated with extracellular matrix (ECM) remodelling and often coexists with myocardial fibrosis (MF); however, the causality of these conditions is not well established.

**Objective:**

We aim to corroborate AF to MF causality by quantifying left atrial (LA) fibrosis in cardiac magnetic resonance (CMR) images after persistent rapid ventricular pacing and subsequent AF using a canine model and histopathological validation.

**Methods:**

Twelve canines (9 experimental, 3 control) underwent baseline 3D LGE-CMR imaging at 3T followed by insertion of a pacing device and 5 weeks of rapid ventricular pacing to induce AF (experimental) or no pacing (control). Following the 5 weeks, pacing devices were removed to permit CMR imaging followed by excision of the hearts and histopathological imaging. LA myocardial segmentation was performed manually at baseline and post-pacing to permit volumetric %MF quantification using the image intensity ratio (IIR) technique, wherein fibrosis was defined as pixels > mean LA myocardium intensity + 2SD.

**Results:**

Volumetric **%**MF increased by an average of 2.11 ± 0.88% post-pacing in 7 of 9 experimental dogs. While there was a significant difference between paired %MF measurements from baseline to post-pacing in experimental dogs (P = 0.019), there was no significant change in control dogs (P = 0.019 and P = 0.5, Wilcoxon signed rank tests). The median %MF for paced animals was significantly greater than that of non-paced dogs at the 5-week post-insertion time point (P = 0.009, Mann Whitney U test). Histopathological imaging yielded an average %MF of 19.42 ± 4.80% (mean ± SD) for paced dogs compared to 1.85% in one control dog.

**Conclusion:**

Persistent rapid ventricular pacing and subsequent AF leads to an increase in LA fibrosis volumes measured by the IIR technique; however, quantification is limited by inherent image acquisition parameters and observer variability.

## Introduction

Atrial fibrillation (AF), the most common form of cardiac arrhythmia, is characterized by rapid and irregular atrial rhythm. AF is often a precursor of several cardiac and cerebrovascular complications, including ischemic stroke, transient ischemic attack, and heart failure [1–3]. Extracellular matrix (ECM) remodelling is an important contributor to the AF substrate, and thus, AF often co-exists with interstitial myocardial fibrosis (MF) [4, 5]. The underlying pathophysiological factors that contribute to AF are yet to be fully understood. ECM remodelling is known to contribute to the onset and progression of AF [4–6]. Likewise, atrial ECM remodelling is known to create re-entry pathways for abnormal electrophysiological activity, which can perpetuate AF [7–10]. However, ECM remodelling may also occur as a consequence of a prior fibrillation process [4, 5, 7]. While MF is frequently observed in AF, a definitive causal relationship has yet to be established.

Detection left atrial (LA) MF via late gadolinium enhanced (LGE) cardiac magnetic resonance (MR) has been of increasing interest for pre- and post-ablation evaluation [11], indicating the need for objective analysis methods for quantification of LA MF. The image intensity ratio (IIR) analysis method [11–14] defines LA fibrosis based on IIR thresholds after normalizing LA myocardial intensity using the mean intensity of the blood pool volume as reference. This method mitigates signal intensity variability caused by intrinsic physiological factors, therefore producing more consistency in atrial fibrosis quantification. The method has been validated at 1.5T and 3T [11, 13, 15], but has not yet been applied in a serial imaging study with histological validation. For LA fibrosis quantification to be translated into clinical practice, further validation of quantification methodologies and thresholds are required. Therefore, the goal of this study was to validate the IIR method for atrial MF quantification after rapid ventricular pacing using a canine model and histopathology.

## Methods

### Study group and animal use protocol

**Fig 1:**
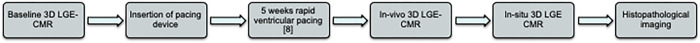
Flow-chart of study protocol and 3D LGE-CMR imaging time points. Six-step study protocol underwent by experimental canines. Control canines underwent the same sequence of events excluding the 5 weeks of rapid ventricular pacing (i.e., the third step in the flow-chart).

Twelve (12) canines (9 experimental, 3 control) underwent serial 3D LGE-CMR imaging at 3T using a Siemens Biograph PET-MR system (Siemens Healthineers, Erlangen, Germany). Imaging was performed at sequential time points, including baseline (i.e., before pacing), in-vivo (after pacing) and in-situ (post-mortem) under general anesthesia, which was induced by propofol and maintained with isoflurane. A flow-chart of the study protocol is demonstrated in Fig 1.

All canines used in this study were female, bred-for-research adult hounds weighing an average of approximately 20 kg. Vitals of all animals, including capillary refill time (CRT), heart rate, respiratory rate, hydration level and mucous membrane color were monitored daily to assess animal pain/discomfort for the duration of this study. Due to the nature of this study wherein two groups of animals were compared, investigators were aware of which animals were controls and which were not. However, emphasis was put on treating the animals equivalently at each stage in the pacing model and during CMR imaging. Animal Use Protocol (AUP) and ethics approval was provided by the institutional Animal Care Committee (ACC) in accordance with the Animal Research: Reporting of In vivo Experiments (ARRIVE) essential 10 guidelines [16].

### Image acquisition and pacing model

3D LGE-CMR imaging was acquired using a spoiled 3D inversion recovery gradient echo pulse sequence (“3D IR-FLASH”) [17, 18] approximately 7–15 minutes post injection of 0.2 mmol/kg gadobutrol (Gadovist, Bayer Healthcare, Mississauga, Ontario, Canada) at the timepoints referred to in Fig 1. Images were acquired using a 32-channel body matrix coil and a 24-channel spine coil. Parallel imaging was employed using the generalized partially parallel acquisition (GRAPPA) method with an acceleration factor of 2. Average sequence parameters for the 3D IR-FLASH sequence are given in Table 1.

**Table 1:** Average 3D IR-FLASH pulse sequence parameters.

Slice thickness (mm)	0.975
Resolution (mm^2^)	0.625
TR (ms)	1.68
TE (ms)	1.34
TI (ms)	341.25
Flip angle (^o^)	19.5
Trigger time (ms)	484.06
Percent sampling (%)	100

The signal to noise ratio (SNR) of the 3D IR-FLASH sequence was computed using the NEMA 4 method [19, 20] in a single slice at each time point across all animals. This technique computes the SNR using a region of interest (ROI) of the blood pool (i.e., the signal) and four rectangular ROIs of the background (i.e., the noise). The SNR was computed by dividing the mean signal intensity of the blood pool ROI by the average of the standard deviations (SDs) of the four noise ROIs, and then multiplying the computed SNR value by a Rayleigh correction factor of 0.66. The mean SNR computed across all animals was 357 ± 203 decibals (dB) at the baseline time point, 169 ± 91 dB at the in-vivo time point, and 331 ± 114 dB at the in-situ time point.

Both control and experimental canines underwent baseline imaging to establish a reference level of MF. A ventricular pacemaker was implanted in all dogs under anesthesia and attached to a pacing lead in the right ventricular (RV) apex as per the canine model developed at the Montreal Heart Institute [21]. In brief, experimental animals underwent 5 weeks of rapid ventricular pacing (220 to 240 bpm) to achieve progressive atrial fibrosis. The animals in the control group underwent surgical insertion of the pacing device but no rapid ventricular pacing, to assess the effect of surgical insertion on LA enhancement and MF.

Following the 5-week period, the pacing device was removed to perform 3D LGE imaging in-vivo. Canines were then euthanized via overdose of potassium chloride while under general anesthesia (induced by propofol and maintained by isoflurane) and 3D LGE imaging was repeated in-situ. A second dose of 0.2 mmol/kg gadolinium was administered 5 minutes prior to sacrifice to obtain in-situ (post-mortem) images, which were used to aid in LA segmentation, as they are uncorrupted by the physiological artifacts encountered on in-vivo imaging. Finally, the hearts were excised, and histopathological imaging was performed. The data underlying in this article (i.e., LGE-CMR and histopathology imaging) is available via the Supporting Information.

### Image processing

#### Segmentation of LA myocardium and enhanced regions

**Fig 2:**
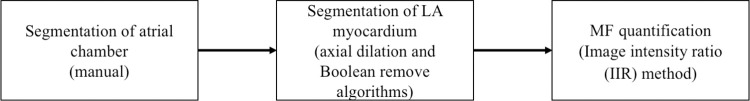
Image processing pipeline. Steps used to quantify MF in 3D IR-FLASH LGE-CMR images at baseline, in-vivo and in-situ time points.

Several image processing steps were performed on the 3D IR-FLASH images at baseline, in-vivo, and in-situ time points, as demonstrated in the pipeline shown in Fig 2.

**Fig 3:**
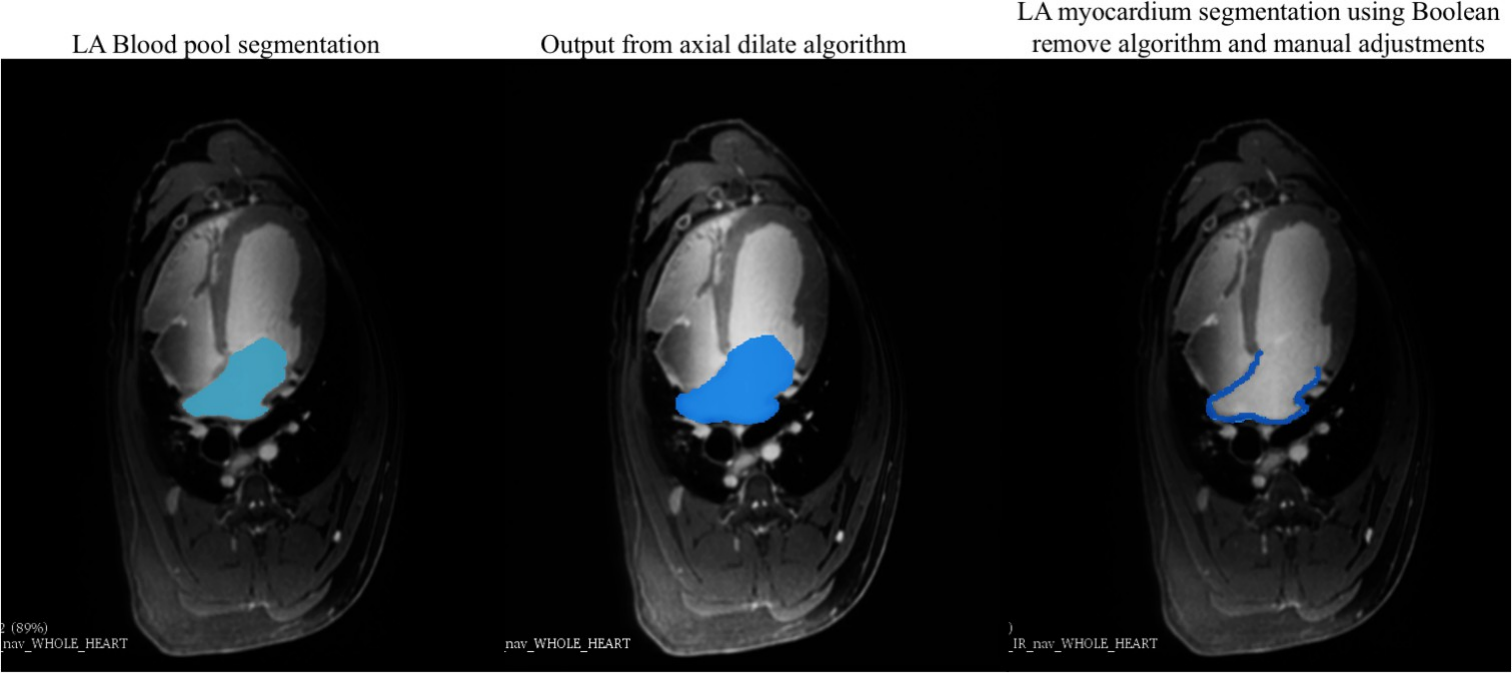
Image processing workflow. Example workflow of LA blood pool and myocardial segmentation using axial dilate and Boolean remove algorithms in in-situ IR-FLASH CMR images. Manual segmentation of the LA blood pool is shown on the left. The axial dilate algorithm expands the blood pool segmentation, as demonstrated in the middle image. The Boolean remove algorithm subtracts the blood pool chamber (left) from the axially dilated segmentation (middle) to reveal the LA wall boundaries. Corrections are done manually to remove erroneous areas as needed, before revealing the final LA myocardial wall segmentation (right).

The LA chamber (i.e., blood pool) was segmented manually by a trained operator (redacted) using the Cardiac MRI Toolkit (Scientific Computing and Imaging Institute, University of Utah, 2019) in 3D Slicer software [22]. To delineate the borders of the LA myocardial wall, axial dilation and Boolean remove algorithms were applied to the LA chamber segmentations. The axial dilate algorithm performs a 4-pixel dilation in the axial plane to expand the manual chamber segmentation into the myocardial wall boundaries. The Boolean remove algorithm subtracts the blood pool mask from the dilated mask, generating a preliminary segmentation of the LA myocardial wall. The operator is required to make manual adjustments as necessary, to ensure non-myocardial tissue regions are erased from the preliminary output (i.e., the region between the LA and RA septum). An example of the execution of this workflow is depicted in Fig 3. This process was performed at each time point, beginning with the in-situ image set. In-situ images allowed for increased accuracy in manual blood pool chamber segmentation since the images were not corrupted by cardiac and respiratory motion. These images served as an anatomical guideline for baseline and post-pacing time points of the same animal.

To quantify MF, the image intensity ratio (IIR) thresholding method [12] was used in order to compare computed fibrosis percentages across different animals. Voxel intensities of the LA myocardium were first normalized by dividing each voxel in the LA by the mean intensity of the LA blood pool, as in Eq 1. An alternative, commonly used normalization technique [23] instead normalizes the LA wall intensities by subtracting the mean intensity of the LA blood pool from raw LA wall intensities and dividing the result by the LA blood pool standard deviation. However, this technique was not appropriate for the canine CMR images, as average blood pool intensities exceeded most LA wall intensities among most canines due to the relatively high gadolinium dose administered, and therefore, excessive brightness in the blood pool.

Normalized left atrial wall intensity NI(x) is given by the IIR normalization technique,

$$NI(x)=\frac{I(x)}{\mu_{BP}},$$ (1)

Where NI(x) is normalized left atrial wall intensity, I(x) is the original intensity and μ_BP_ is the average signal intensity of the left atrial blood pool.

Atrial MF was then quantified using an IIR threshold > mean IIR + 2 IIR SDs (Eq 2). The IIR+2SDs technique was selected after establishing that this threshold yielded the best quantification results for LA enhancement versus other thresholding techniques, including IIR+ 3 or 4 SDs, which exhibited too few pixels with fibrotic signal intensities to quantify, and STRM using a mean reference intensity derived from remote left ventricle (i.e., not the left atrium, in order to mitigate partial volume effect) and 2–4 SDs, which significantly over-prescribed the enhanced LA regions.

Thus, the threshold T_fib_ for LA fibrosis quantification is given by,

$$T_{fib}=\mu_{NI}+2\sigma_{NI},$$ (2)

where μ_NI_ is the mean IIR normalized intensity of the left atrial wall and σ_NI_ is the standard deviation of IIR normalized intensities.

#### Computation of myocardial fibrosis volumes

MF volumes were computed by first finding the sum of all enhanced voxels within the LA myocardial wall and multiplying that sum by the voxel dimensions. We computed the percentage of MF (%MF) in the entire LA myocardium by dividing MF volumes by the overall myocardial wall volume, which was computed using the aforementioned approach. Normalization, IIR thresholding and MF volume quantification were computed using MATLAB 2020a. Resultant %MF are reported only in baseline and post-pacing time points. This is due in large part to the additional dose of gadolinium-based contrast agent administered prior to sacrifice. This, combined with the lack of physiological blood flow caused a pooling and stagnation of gadolinium within the blood pool chamber in the in-situ images, rendering these images inappropriate for IIR %MF computation, since the IIR thresholding technique relies on blood pool-based normalization. The baseline and post-pacing images do not present these complications, and therefore LA enhancement volumes are more reliable at these time points.

The %MF measured across the entire LA myocardium is measured on a 3D volumetric basis, wherein every LA myocardial slice contributes toward the computation, inclusive of image slices where no fibrosis is present. However, validation using histopathological imaging is measured on a 2D slice-wise basis, wherein the %MF is computed across the area of only one imaging slice. Therefore, in order to better corroborate our fibrosis quantification method with histopathology, we also computed the area-wise %MF using the IIR thresholding technique described above in a single slice of the 3D LGE imaging stack. We chose the slice demonstrating the greatest extent of LA enhancement for both baseline and post-pacing time points. Area-wise %MF was computed by taking the sum of all enhanced MF pixels in the slice and dividing by the overall myocardial wall area in that respective slice.

The evolution of LA enhancement was assessed quantitatively and qualitatively across baseline and post-pacing time points. Quantitative assessment was performed by comparing %MF sequentially between baseline and post-pacing images for individual dogs. We also computed the mean and median %MF across experimental and control groups at baseline and post-pacing. In addition, we compute the absolute %MF difference (D_abs_) between post-pacing and baseline time points for all dogs using the following approach,

$$D_{abs}=V_{p}-V_{b}$$ (3)

where V_b_ and V_p_ are the %MF values measured at baseline and post-pacing, respectively. Notably, the absolute difference D_abs_ is not expressed as an absolute value. The mean and median values of D_abs_ were computed and compared between experimental and control dog groups. Qualitative assessment was performed by assessing the spatial distribution of MF between images at all consecutive time points following landmark-based registration, described below.

#### Landmark-based 3D image registration

To localize the progression of fibrosis across time points, a landmark-based rigid registration was performed to mitigate motion differences and any disparities in anatomical positioning/shape between the 3D IR-FLASH at baseline and post-device insertion. Thirteen landmarks were selected within the 3D IR-FLASH imaging stacks at each time point using the Fiducial Registration module in 3D Slicer. These landmarks included the ascending and descending aorta, aortic arch, left and right pulmonary arteries, inferior/superior left and right pulmonary veins, left atrial/ventricular septum, LA appendage, left anterior descending artery, and aortic valve. Fixed landmarks were designated in the post-pacing images, while baseline and in-situ fiducials were chosen as moving landmarks (i.e., the resultant transform maps fiducials from the baseline/in-situ space to the fixed landmarks in post-pacing space). In rigid registration, the resultant transform minimizes the sum of squared differences between fixed and moving landmarks.

The landmark-based registration technique allowed myocardial volumes to be rendered in the same space for qualitative assessment across time points. However, inherently, myocardial positioning and/or shape will change among images acquired post-operatively at different time points. Thus, quantitative overlap metrics were not computed on MF volumes across different time points.

#### Histopathological imaging

Histopathological imaging of the LA was performed on a slice-by-slice basis in six of the nine experimental canines to permit fibrosis quantification. Immediately after sacrifice, canine hearts were excised and immersed in formalin fixative for a minimum of 7 days, after which, the LA was isolated for tissue analysis. The LA was embedded in paraffin to allow for slice-by-slice analysis. Paraffin blocks were cut every 5 mm through the LA, and each block had sequential slices cut 4 μm thick using a large-scale microtome. Slices were put on positivity charged slides and deparaffinized prior to staining with Masson’s Trichrome. The Masson’s Trichrome staining technique was selected as it is highly accessible, reliable, and has been validated for identification of collagen among numerous studies concerning heart disease [24–27]. After staining, slides were imaged on brightfield mode with a Tissuescope LE (Huron Digital Pathology) on 20x magnification. Images were created as non-proprietary 24-bit RGB Pyramidal Big TIFF with JPEG 2000 compression. Between 2 and 7 slices were acquired per canine in the LA. Fibrosis was quantified using FIJI v. 1.49v software (National Institutes of Health, Maryland, USA http://imagej.nih.gov) and analyzed using a script that quantifies the percentage of fibrotic tissue in each sample by setting thresholds for fibrosis (blue) and nonfibrotic tissue (red). These thresholds are optimized across each sample in the study to normalize fibrosis quantification.

### Method evaluation and statistical analysis

#### Observer variability

To validate our MF quantification technique, we performed an inter- and intra- observer variability analysis using two operators (redacted). Due to resource and time restraints, we randomly selected three animals (2 experimental, 1 control) for this analysis. Each operator performed the MF segmentation as per the pipeline in Fig 2 three times at each time point per animal. To assess the agreement of overlap of segmentation volumes across operators, we computed the dice similarity coefficient (DSC), expressed as a percentage between 0% (no regional overlap) to 100% (complete regional overlap) for the blood pool chamber, LA myocardial wall and MF regions across each operator’s three segmentation attempts. We also computed the DSC metric on the aforementioned regions among all attempts between both operators. The definition of DSC is given in Eq 1 in S1 Appendix. In addition to computation of the DSC, we also used the blood pool chamber, LA myocardial wall and MF volumes per each attempt/operator to compute boundary F1 score, precision, recall, and relative volume error (see Eqs 2, 3.1 and 3.2 in S1 Appendix). Lastly, we computed and compared the volumetric %MF across the entire LA myocardium for each of the operators’ three attempts and for each time point.

#### Statistical tests

Non-parametric statistics were computed in this study due to the small dataset size. To assess the significance between baseline and post-pacing MF measurements within experimental and control groups, we used the Wilcoxon signed rank test on paired %MF measurements within respective groups.

To compare differences between post-pacing %MF measurements computed among experimental versus control groups, we used the Mann Whitney U test.

Intra-observer variability of %MF was evaluated using the intraclass correlation coefficient ICC on %MF calculated from the operators’ three segmentation attempts. The calculation for ICC is given as follows:

$$ICC={\left(1+\frac{\sigma_{w}^{2}}{\sigma_{b}^{2}}\right)}^{-1}$$ (4)

where σ^2^_w_ is the variance within measurements reported by one operator and σ^2^_b_ is the variance between measurements reported by both operators [28].

Inter-observer variability of %MF was evaluated using the inter-rater reliability metric R, given by:

$$R={\left(1+\frac{\sigma_{w}^{2}}{{k\sigma }_{b}^{2}}\right)}^{-1}$$ (5)

where σ^2^_w_ and σ^2^_b_ are defined as denoted in (4) and k indicates the number of operators [28]. The ICC and R metrics range between 0 and 1, such values closer to 0 denote poor agreement (high variation) between observations and values closer to 1 denote high agreement (low variation) between observations.

## Results

### Segmentation of MF volumes using IIR Thresholding in 3D-FLASH CMR

The IIR threshold used to define fibrosis ranged between 0.90–1.3 across all time points for each dog. Among paced animals, average IIR thresholds between 1.10 ± 0.12 at baseline and 1.0 ± 0.05 post-pacing were applied (expressed as average IIR threshold ± SD). Among the three control dogs, average IIR thresholds of 1.10 ± 0.06 and 1.15 ± 0.07 were applied at baseline and post device-insertion time points, respectively.

MF volumes computed using the IIR technique were quantified in cubed millimetres and expressed as a percentage of the overall LA myocardial wall (%MF) in Table 2. We computed an average %MF of 1.27 ± 1.10% at baseline and 2.80 ± 0.98% post-pacing across all experimental dogs (expressed as mean ± SD). In control dogs, an average %MF of 0.69 ± 0.83% was computed at baseline versus 0.93 ± 0.20% post-surgery (mean ± SD).

**Table 2:** Volumetric %MF measurements computed in 3D IR-FLASH CMR images among experimental and control animals.

		Baseline %MF over total LA myocardial volume	Post-pacing %MF over total LA myocardial volume	Absolute difference (D_abs_)
Experimental	Mean ± SD	1.27 ± 1.10%	2.80 ± 0.98%	1.53 ± 1.42%
	Median ± IQR	0.99 ± 2.08%	2.96 ± 1.64%	1.82 ± 2.24%
Control	Mean ± SD	0.69 ± 0.83%	0.93 ± 0.20%	0.23 ± 0.65%
	Median ± IQR	0.39 ± 1.2%	0.95 ± 0.30%	0.56 ± 0.89%

LA fibrosis volumes computed using the IIR technique are expressed as a percentage of MF (%MF) across the overall LA myocardial volume and are shown at baseline and post-pacing (i.e., post device-insertion for the control group) time points. Average and median values of %MF and D_abs_ are demonstrated across the experimental and control groups as mean volume ± standard deviation (SD) and median volume ± interquartile range (IQR), respectively.

Median %MF values (expressed as median ± IQR) of 0.99 ± 2.08% versus 2.96 ± 1.64% were reported in experimental dogs at baseline and post-pacing, respectively. Median %MF values of 0.39 ± 1.2% versus 0.95 ± 0.30% (median ± IQR) were computed for control dogs at baseline and post device-insertion time points, respectively.

**Fig 4:**
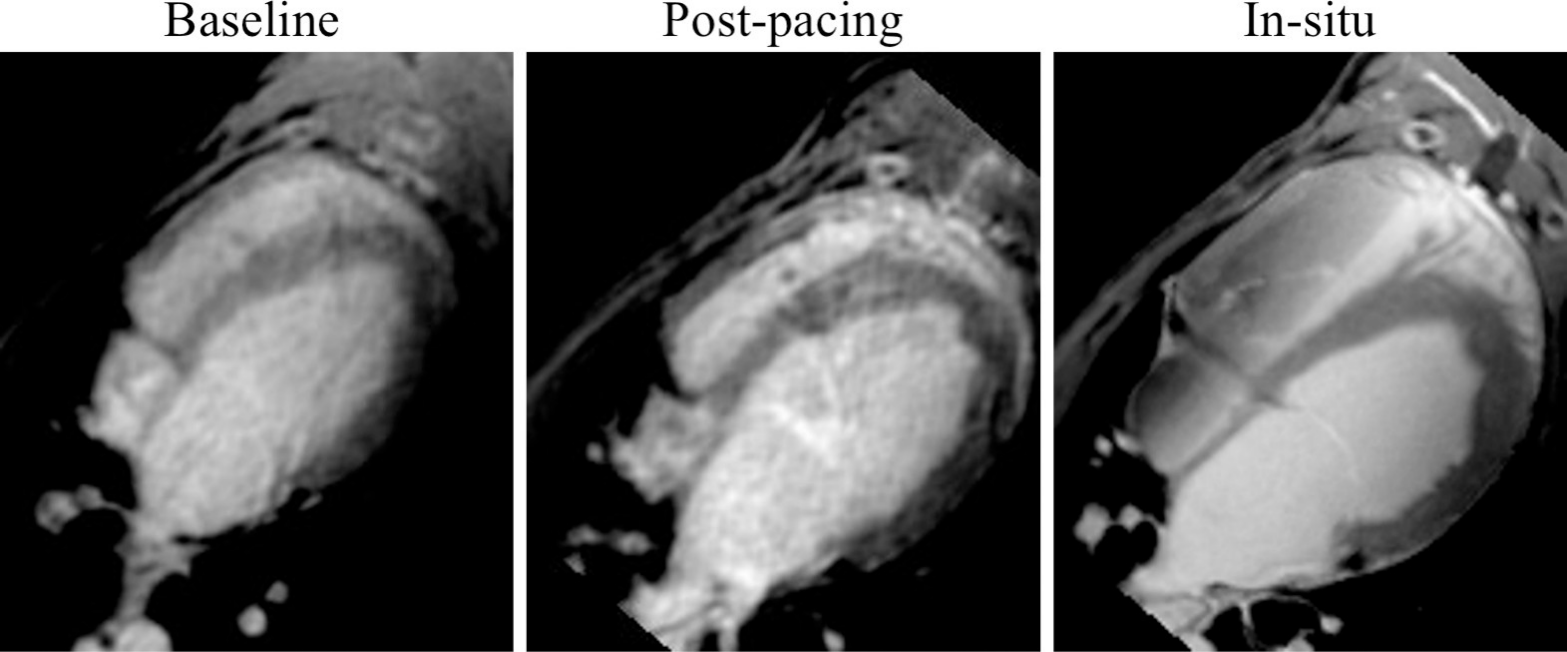
LA enhancement demonstrated in 3D LGE CMR at baseline, post-pacing (in-vivo) and post-mortem (in-situ). A single slice from 3D IR-FLASH LGE CMR acquisitions are demonstrated at baseline (left column), post-pacing (middle) and in-situ (right column) in experimental dog 7 at approximately the same slice location. LA enhancement is demonstrated in the anterior aspect of the LA in post-pacing and in-situ images. Post-pacing images are significantly corrupted by motion artifact due to irregular, elevated heart rate that occurred as a result of pacing.

The average absolute difference D_abs_ computed between baseline and post-pacing time points was 1.53 ± 1.42% across the experimental group and 0.23 ± 0.65% across the control group (mean ± SD). The median D_abs_ computed between baseline and post-pacing time points was 1.82 ± 2.24% versus 0.56 ± 0.89% among the experimental and control animals, respectively (median ± IQR). Fibrosis quantification results at baseline and post-pacing time points are demonstrated on a per-animal basis in S1 Table 1 in S1 Appendix. Example CMR images at each time point for experimental dog 7 are demonstrated in Fig 4.

**Fig 5:**
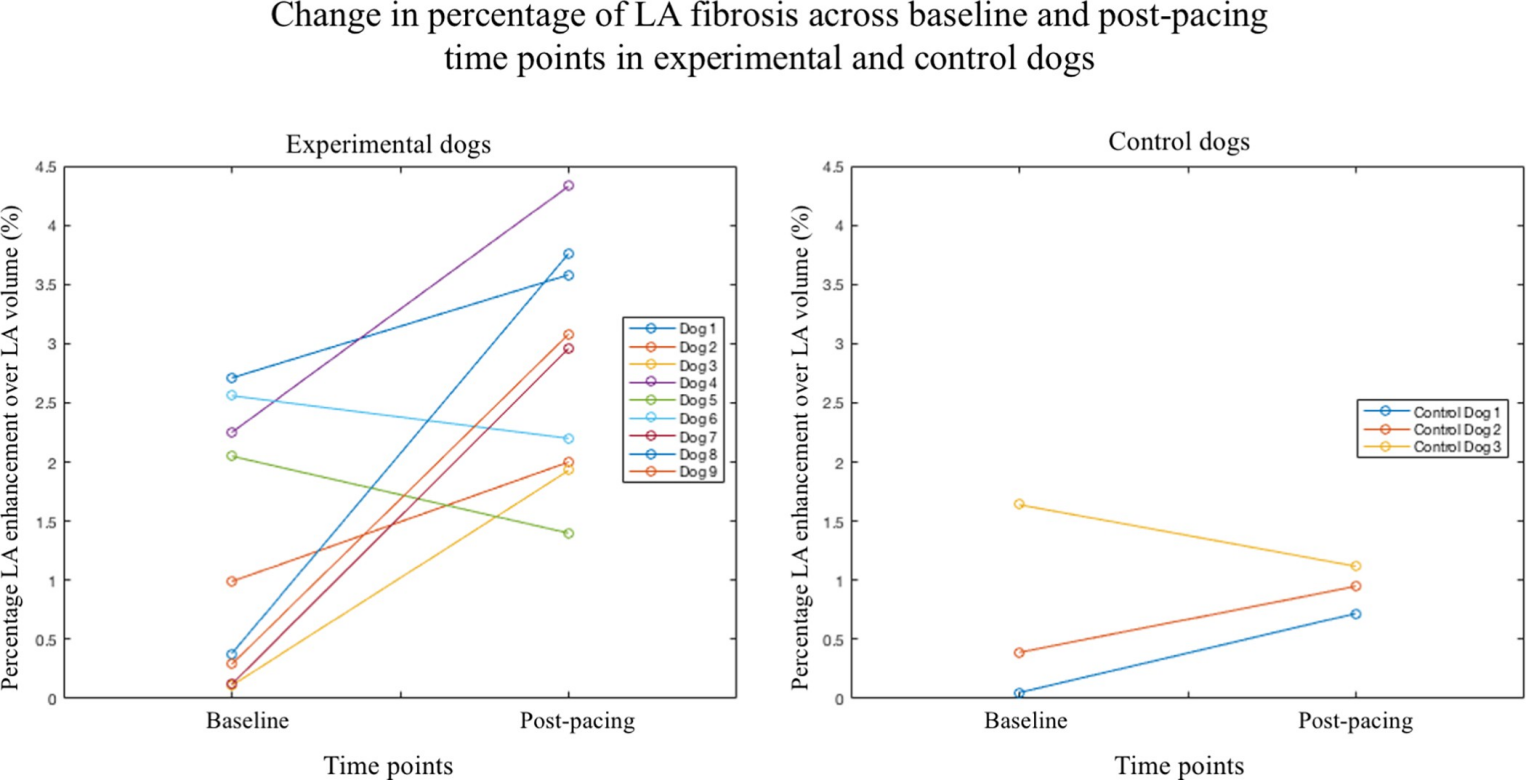
Dot and line plot of %MF changes across time points. Dot and line plot demonstrating the change in percent fibrosis (%MF) over the LA volume for experimental (paced) dogs (right) and control dogs (left) on a per-animal basis. Baseline and post-pacing time points are indicated on the x-axis while LA fibrosis percentage is demonstrated on the y-axis.

Fig 5 demonstrates a dot and line plot showing the change in volumetric %MF for each animal in experimental and control groups across baseline and post device-insertion time points. An increase in the reported %MF was demonstrated in seven of nine experimental dogs (i.e., paced dogs 1, 2, 3, 4, 7, 8 and 9) across baseline to post-pacing time points (see S1 Table 1 in S1 Appendix). The mean D_abs_ computed across these seven animals was measured at 2.11 ± 0.95% (mean ± SD). Across the seven dogs with increased LA enhancement, the median D_abs_ demonstrated an increase of 2.08 ± 1.62% (median ± IQR). Dogs 5 and 6 demonstrate a decrease in %MF of 0.65% and 0.36%, respectively, from baseline to post-pacing.

Control dog 3 demonstrated a decrease in %MF from baseline to post device-insertion, while control dogs 1 and 2 demonstrate an increase in %MF. However, the increase in %MF between baseline and post device-insertion that is exhibited in control dogs 1 and 2 is less than 0.67%, compared to an increase of 2.11% (mean) in experimental dogs. A Wilcoxon signed rank test demonstrated statistically significant differences in volumetric %MF between baseline and post-pacing time points for the experimental group (i.e., P = 0.019); no significant difference in volumetric %MF was observed in the control group (P = 0.50). Additionally, post-pacing and post-device insertion volumetric fibrosis measurements were compared between experimental and control dogs. A Mann-Whitney U-Test demonstrated a statistically significant difference in %MF measurements for paced vs. non-paced dogs at the 5-week post-insertion time point (two-tailed P-value = 0.009).

### Segmentation of slice-wise MF areas using 3D-FLASH CMR

Table 3 demonstrates slice-wise %MF at baseline and post-pacing and D_abs_ measurements computed in experimental and control animals. We report average 2D slice-wise %MF (expressed as mean ± SD) of 5.48 ± 3.84% and 14.38 ± 9.96% in experimental animals at baseline and post-pacing time points, respectively. In control dogs, the average area-wise %MF was computed at 9.89 ± 0.61% and 10.26 ± 1.15% at baseline and post-pacing time points. We report an average absolute difference D_abs_ of 8.90 ± 8.59% in experimental animals versus 0.37 ± 0.94% in control animals (mean ± SD).

**Table 3:** Slice-wise %MF measurements computed in 3D IR-FLASH CMR images among experimental and control animals.

		Baseline %MF over single LA myocardial slice area	Post-pacing %MF over single LA myocardial slice area	Absolute difference (D_abs_)
Experimental	Mean ± SD	5.48 ± 3.84%	14.38 ± 9.96%	8.90 ± 8.59%
	Median ± IQR	6.04 ± 6.39%	11.22 ± 12.95%	7.22 ± 11.73%
Control	Mean ± SD	9.89 ± 0.61%	10.26 ± 1.15%	0.37 ± 0.94%
	Median ± IQR	9.98 ± 0.92%	9.98 ± 1.68%	0.74 ± 1.32%

Slice-wise (i.e., area-wise) LA fibrosis measurements are expressed as a percentage of MF (%MF) across the overall LA myocardial area on the slice with which the greatest extent of LA enhancement was shown. Baseline and post-pacing (i.e., post device-insertion for the control group) slice-wise %MF measurements are reported across the experimental and control groups. Average and median values of %MF and D_abs_ are expressed as mean area ± standard deviation and median volume ± interquartile range (IQR), respectively.

Median %MF values (median ± IQR) of 6.04 ± 6.39% versus 11.22 ± 12.95% are reported in experimental dogs at baseline and post-pacing time points, respectively, yielding a median absolute difference D_abs_ of 7.22 ± 11.73%. Among control animals, median %MF values of 9.98 ± 0.92% versus 9.98 ± 1.68% are reported at baseline and post device-insertion time points, respectively, yielding a median absolute difference D_abs_ of 0.74 ± 1.32% (median ± IQR).

Per-animal %MF measurements in the 2D slice demonstrating the greatest extent of LA enhancement are demonstrated in S1 Table 2 in S1 Appendix for each time point on a subject-by-subject basis. Like the volumetric %MF measurements demonstrated in S1 Table 1 in S1 Appendix, an increase in the reported area-wise %MF was demonstrated in the same seven of nine experimental dogs across baseline to post-pacing time points, wherein dogs 5 and 6 demonstrate a decrease in the absolute area-wise %MF difference (D_abs_), of 0.91% and 3.24%, respectively, from baseline to post-pacing.

In our 2D slice-wise analysis, the Wilcoxon signed rank test demonstrated a statistically significant difference in experimental dogs paired %MF measurements at baseline versus post-pacing time points (i.e., P = 0.0195), but failed to show significance in control dogs %MF measurements from baseline to post device-insertion time points (i.e., P = 0.50). A Mann-Whitney U test did not demonstrate statistically significant differences between area-wise %MF measurements in experimental dogs versus control dogs (P = 1.00).

### Registration of myocardial volumes

**Fig 6:**
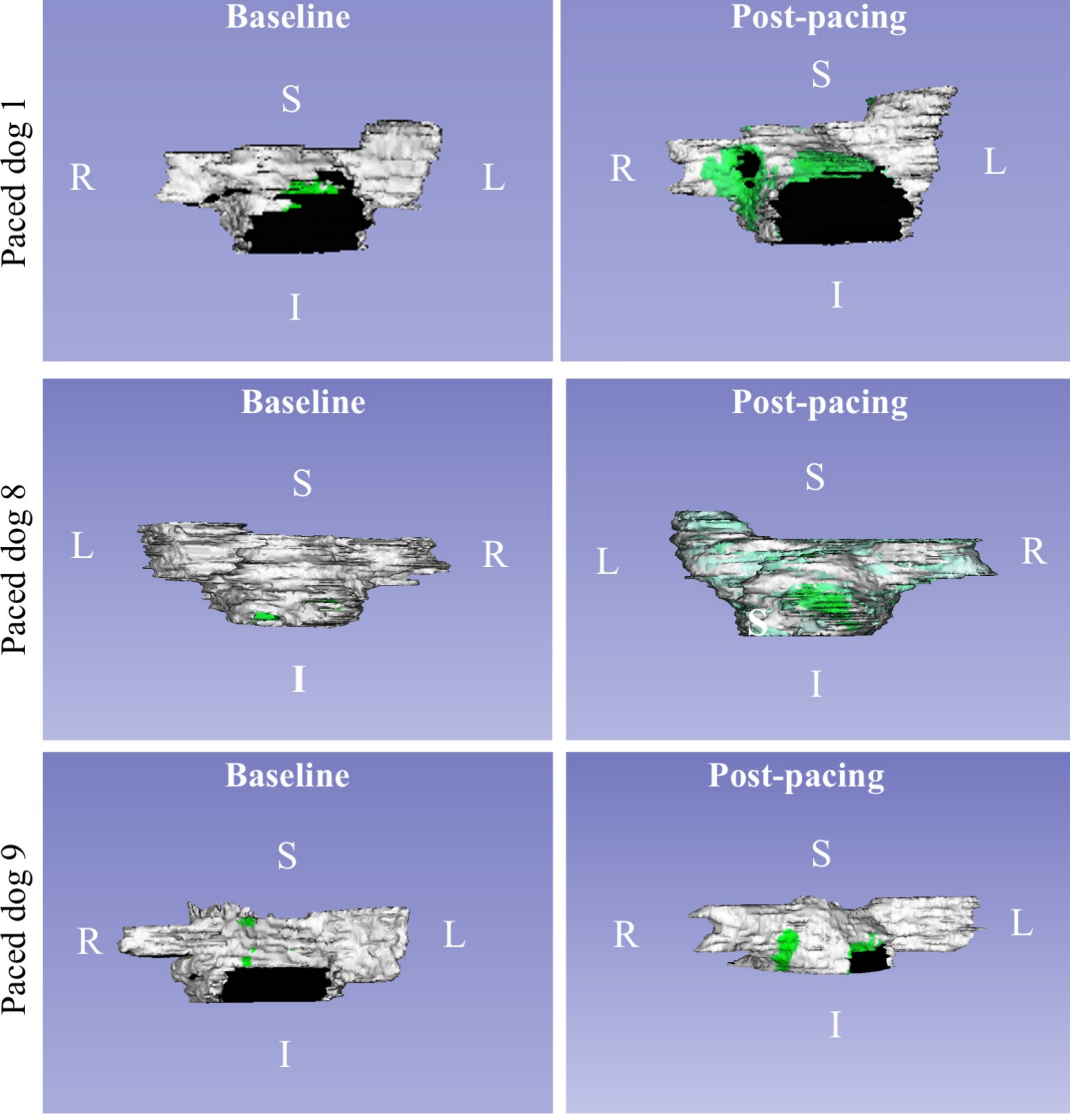
Rendered 3D volumes of LA myocardium and enhancement regions in experimental animals. Evolution of LA enhancement volumes in three example dogs at baseline (left), post-pacing (middle) and in-situ (right). The LA myocardial volume is rendered in white color, while enhanced regions are depicted in green. The black regions demonstrate regions outside of the myocardium, such as the septum space between the left atrium to left ventricle (such as in Paced dog 9).

**Fig 7:**
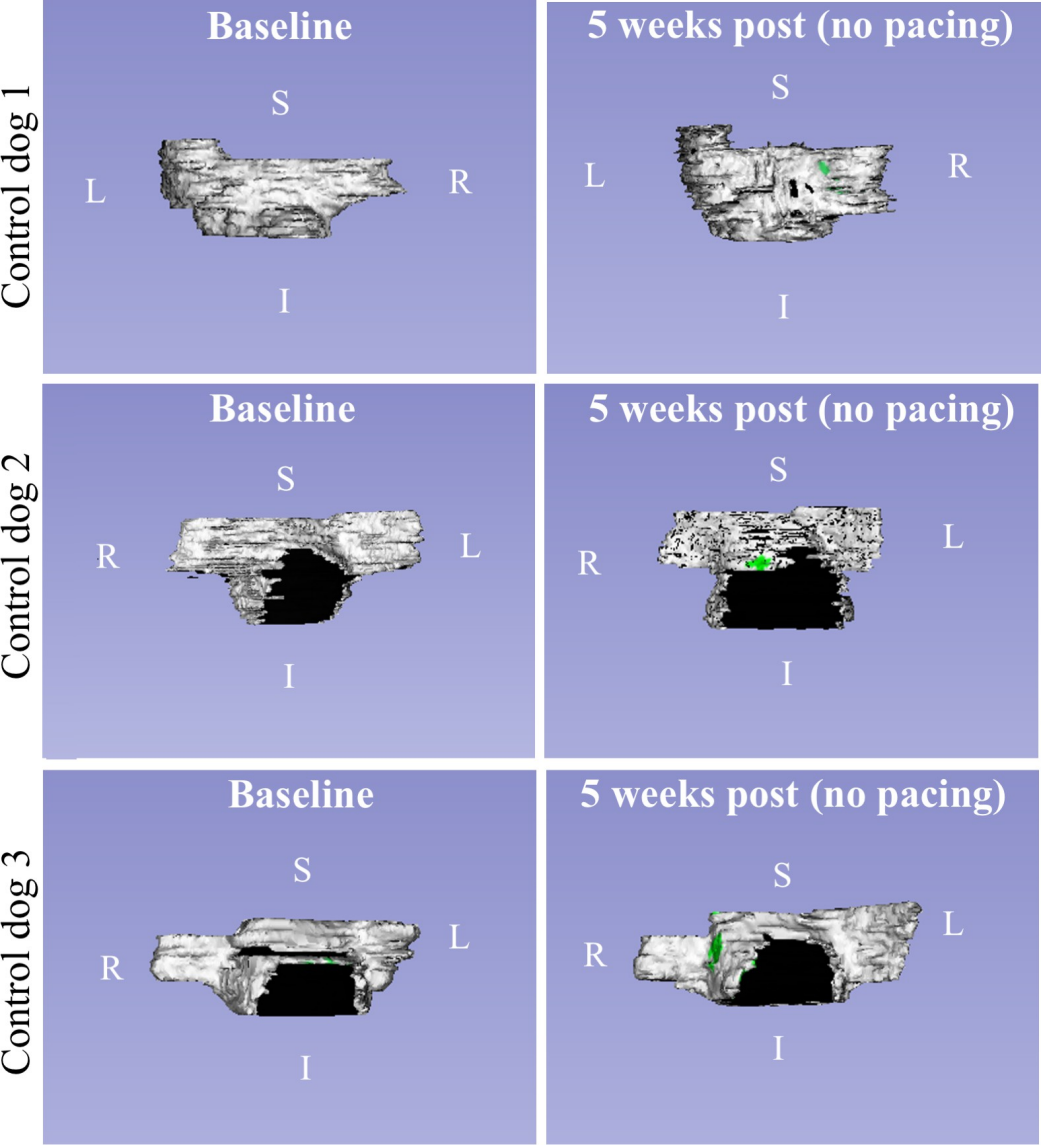
Rendered 3D volumes of LA myocardium and enhancement regions in control animals. Evolution of LA enhancement volumes in control dogs at baseline (left) and 5 weeks post insertion of pacing device (right) with no rapid ventricular pacing. In-situ 3D-IR-GRE MR images were not acquired in these canines.

Landmark-based image registration was used to qualitatively assess LA enhancement distribution among canines across all time points. Fig 6 demonstrates the evolution of LA enhancement across time points after landmark-based registration in three example dogs. Evolution of LA enhancement in control dogs is demonstrated in Fig 7.

### Histopathological validation

Fibrosis regions were quantified in a voxel-by-voxel basis in histopathological images and are expressed as a percentage of the myocardium per slice in Table 4. LA fibrosis volumes ranged between 8.74–33.18% of myocardium per histopathological slice in the paced dogs. Among the five paced dogs, we report a mean of 19.42 ± 4.80% (mean ± SD) and median of 20.77 ± 3.92% (median ± IQR) across the reported average percentages of slice-wise MF per animal. Taking the control dog into account, we report an average %MF of 16.48 ± 8.36% (mean ± SD) and median %MF of 20.47 ± 10.06% (median ± IQR) across all six dogs with which histopathological imaging was acquired.

**Table 4:** Left atrial fibrosis volumes computed from histopathological images.

*Experimental dogs*								
Slice #	1	2	3	4	5	6	7	Average
**Dog 1**	N/A	32.43%	23.43%	N/A	13.09%	12.77%	19.17%	20.18%
**Dog 2**	29.51%	23.38%	25.63%	19.35%	18.53%	17.36%	14.83%	21.23%
**Dog 3**	N/A	N/A	N/A	22.17%	19.37%	N/A	N/A	20.77%
**Dog 5**	N/A	N/A	N/A	N/A	N/A	14.28%	33.18%	23.73%
**Dog 6**	N/A	8.99%	12.87%	8.74%	11.69%	13.57%	N/A	11.17%
***Control dogs***								
**Control 3**	3.06%	2.55%	2.24%	0.34%	1.08%	N/A	N/A	1.85%

Histopathological imaging was acquired in 5 experimental and 1 control canine. Regions of fibrosis are expressed as a percentage with respect to healthy myocardium for each slice.

**Fig 8:**
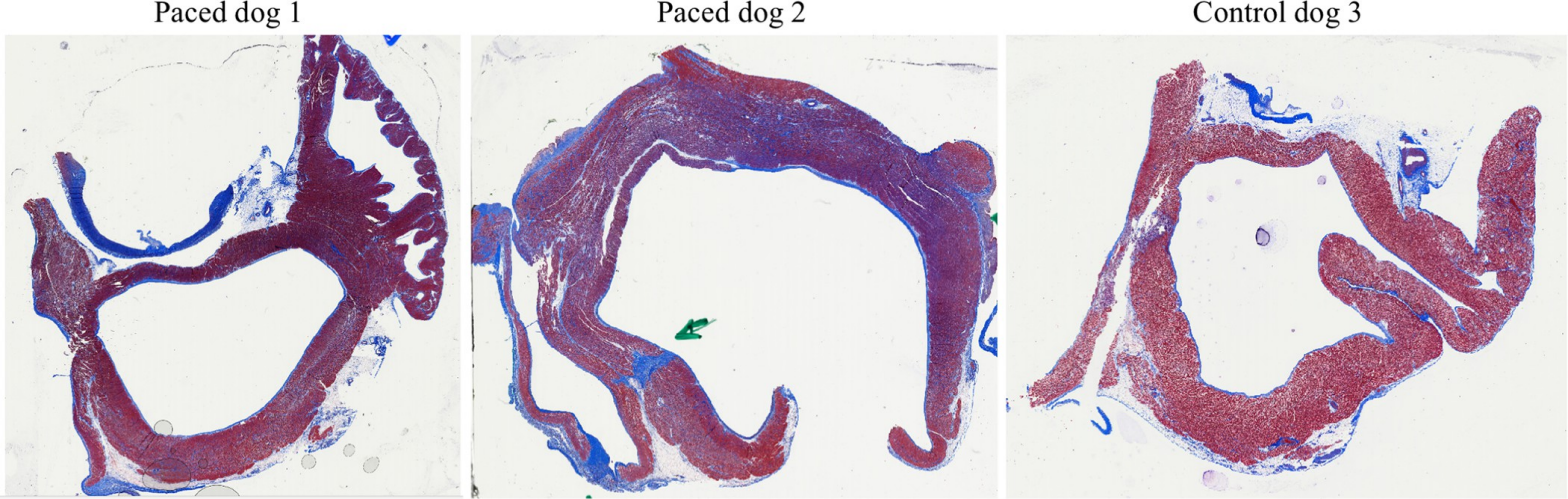
Histopathological imaging of experimental (2) and control animals (1). Example histopathological images of left atrial samples acquired after Masson’s Trichrome staining. Fibrotic areas are shown in blue while normal tissue is shown in red.

Six histopathological slices were acquired from one control dog, with computed MF percentages ranging between 0.34–3.06%. The average MF volume computed across histopathological slices acquired from the control dog was 1.85%. Histopathological images (Masson’s Trichrome stain) corresponding to two experimental and one control dog are shown in Fig 8.

### Observer variability

Table 5 demonstrates the average three-way DSC computed on three segmentations of the myocardial chamber (blood pool), myocardial LA wall and MF volumes across two operators (redacted) in our intra-/inter- observer variability analysis. Likewise, variability results of boundary F1 score, precision, recall and relative volumetric error metrics are reported across each user in S1 Tables 3–6 in S1 Appendix. The volumetric %MF computed per the three segmentation attempts and the mean %MF across all segmentation attempts for each operator is depicted in S1 Table 7 in S1 Appendix.

**Table 5:** Average DSC computed on three segmentation attempts performed by two operators.

		Paced dog 1		Paced dog 3		Control dog 3	
**Operator 1**	**Region**	**Baseline**	**Post-pacing**	**Baseline**	**Post-pacing**	**Baseline**	**Post-pacing**
	DSC Blood pool (%)	88.37 ± 2.09	85.51 ± 1.87	92.13 ± 1.01	89.61 ± 0.82	91.72 ± 1.00	90.49 ± 2.24
	DSC LA wall (%)	49.34 ± 13.57	45.03 ± 7.15	66.01 ± 7.65	64.23 ± 3.02	67.05 ± 3.43	69.49 ± 3.28
	DSC MF volume (%)	14.64 ± 19.52	15.16±20.37	9.80 ± 16.98	20.23 ±30.55	6.84 ± 9.89	8.88 ± 14.26
**Operator 2**	DSC Blood pool (%)	90.40 ± 1.63	91.08 ± 0.08	94.51 ± 1.08	91.96 ± 1.71	94.57 ± 0.10	94.75 ± 0.15
	DSC LA wall (%)	51.81 ± 11.21	54.94 ± 3.97	65.58 ± 7.96	65.55 ± 5.58	69.75 ± 2.61	71.45 ± 1.52
	DSC MF volume (%)	17.04 ± 12.63	24.48 ± 5.24	15.80 ± 12.15	7.88 ± 3.43	25.53 ± 10.81	38.80 ± 11.58
**Operator 1 vs. Operator 2**	DSC Blood pool (%)	88.56 ± 1.21	87.43 ± 4.43	90.84 ± 1.77	88.06 ± 1.56	81.64 ± 0.80	90.79 ± 0.90
	DSC LA wall (%)	65.81 ± 23.90	64.91 ± 21.05	58.23 ± 5.77	55.80 ± 3.19	59.89 ± 1.88	63.62 ± 1.73
	DSC MF volume (%)	34.26 ± 19.91	25.28 ± 21.58	0.48 ± 0.67	0.28 ± 0.46	2.52 ± 2.71	14.58 ± 12.65

Each segmentation attempt is referred to as “segmentation 1, 2 or 3”. The DSC was computed on each operator’s segmentation 1 versus segmentation 2, segmentation 2 versus segmentation 3, and segmentation 3 versus segmentation 1, wherein the average value is reported as a percentage (mean ± SD).

Mean DSC metrics computed across the blood pool chamber demonstrate high agreement in both intra-observer and inter-observer comparison, i.e., >81% reported across both time points for all dogs among both operators. However, accuracy declines as the segmentation becomes focused to smaller regions. For the LA myocardial wall, DSC ranged from 45.03 to 69.49% for Operator 1 and from 51.81 to 71.45% for Operator 2 across all animals and time points in the intra-observer analysis. Among both operators in the inter-observer analysis, the DSC for the LA myocardial wall ranged from 55.80 to 65.81% across all animals and time points. The DSC agreement was found to be lowest among MF volumes in both intra- and inter-observer comparison. We report MF volume DSC metrics in the range of 8.88–20.23% for Operator 1, 7.88–38.80% for Operator 2, and 0.28–34.26% across both operators.

For all segmentation sessions, the %MF measured by Operator 1 showed an increase from baseline to post-pacing time points in both paced animals. Like the reported MF volumes in S1 Table 1 in S1 Appendix, all segmentation attempts by Operator 1 showed a decrease in %MF from baseline to post-pacing in control dog 3.

Likewise, the %MF measurements resulting from Operator 2 demonstrated an increase across all segmentation attempts from baseline to post-pacing in paced dog 3 and two of three attempts for paced dog 1. However, the %MF measurements from Operator 2 for control dog 3 conflicted, with one decreasing and the other increasing from baseline to post-pacing. Notably, a there was an increase in %MF for control dog 3’s post-pacing time point (attempt 2) by Operator 2.

The ICC and R metrics for the %MF are depicted in Table 7 in S1 Appendix. The %MF computed by Operator 1 demonstrated moderate to high agreement across baseline and post-pacing time points, with ICC ranging between 0.45–0.91 across all dogs and all time points. The strongest agreement was observed for post-pacing %MF measurements in control dog 3 and weakest in post-pacing measurements in paced dog 3 for Operator 1.

Operator 2 also demonstrated moderate to high agreement in %MF measurements computed across all dogs and all time points, with ICC values ranging between 0.40–0.93. The strongest agreement in %MF measurements was observed for the baseline timepoint for control dog 3, while the lowest agreement corresponded to the post-pacing timepoint in paced dog 1 for Operator 2. The ICC of absolute %MF differences (D_abs_) also demonstrates moderate to high agreement, ranging from 0.48–0.88 and 0.49–0.99 for Operators 1 and 2, respectively.

Overall, moderate to high agreement was reflected by the inter-rater reliability metric (R), which ranged between 0.58–0.92. The lowest inter-observer agreement was demonstrated at baseline for paced dog 1 and highest agreement corresponded to the baseline for control dog 3. On absolute %MF differences ((D_abs_), moderate to high agreement was also demonstrated, ranging between 0.66–0.82.

## Discussion

In this study, we aimed to validate an objective method for left atrial MF quantification using confirmation from histopathological images and elucidate the relationship between AF and MF in a canine model. We used the IIR method with a threshold of IIR+2SDs to define fibrosis in 9 experimental and 3 control dogs. Overall, a trend of increasing fibrosis was observed across sequential time points. LA enhancement increased from baseline to post-pacing in 7 out of 9 experimental dogs. In control dogs, a marginal increase was demonstrated on average across baseline to post device-insertion points but was negligible compared to the trend observed in animals who underwent pacing.

Two animals in the experimental group demonstrated a decrease in LA enhancement from baseline to post-pacing time points. This decrease in enhancement was less than 1% in both cases and may reflect differences in gadolinium dosing or timing of image acquisition in those animals. Alternatively, an increase in the enhancement threshold may reverse this trend. For example, one animal demonstrated a decrease from baseline to post-pacing time points using the IIR+2SDs threshold; however, an increase in enhancement was demonstrated in the same dog when using a threshold of IIR+3SDs.

Histopathological imaging was acquired in several samples for five experimental dogs and one control dog. Fibrosis on LGE CMR imaging was identified and quantified and compared with histological evidence of fibrosis. Histopathological analysis demonstrated significantly higher fibrosis measurements among experimental animals compared to the control animal. Dogs 5 and 6, which demonstrated a decrease in LA enhancement measurements across baseline to post-pacing CMR images, exhibit confirmed fibrosis in Masson Trichome-stained slides. Thus, the discrepancy in the increasing trend from baseline to post-pacing demonstrated in these dogs through LGE-CMR is likely artifactual, possibly attributable to partial volume effect. However, this is an important consideration demonstrating the difficulty of measuring the extent of LA fibrosis using LGE-CMR alone.

Notably, there is a discrepancy in volumetric %MF measured in post-pacing time points as compared to histopathology. Certain limitations contribute to this discrepancy, namely, coarse spatial resolution of LGE images, partial volume effect, and the fact that volumetric %MF quantifies scar across the entire LA myocardial volume as opposed to a single slice area as with histopathology. Thus, we computed a slice-wise area-wise %MF for better comparison against histopathology. For example, in paced dog 1, we report a volumetric extent of 3.59%MF post-pacing across the entire LA myocardium (i.e., all image slices). However, in the same dog at the post-pacing time point, the slice containing the highest number of MF pixels yields a computed area-wise extent of 34.46% MF, which is in better keeping with the %MF reported in histopathological imaging.

Aside from validating an objective method for MF quantification and corroborating the relationship between AF and MF, this study also highlights the impact of arrhythmia on the capability of LA fibrosis assessment using LGE-CMR. As a result of the 5 weeks of rapid ventricular pacing, the experimental animals developed persistent atrial fibrillation, characterized by abnormally rapid heart rates. Therefore, paced animals exhibited tachycardia during CMR image acquisition, which impacted both image contrast and image quality. It is particularly challenging to select an inversion time that is long enough to result in satisfactory nulling of the myocardium when R-R intervals are short due to fast heart rates. Thus, enhanced LA regions in post-paced LGE CMR images demonstrated suboptimal contrast with healthy myocardium, potentially confounding the computed MF volumes due to partial volume effect. Consequently, a threshold of mean IIR + 2 standard deviations was chosen in order to adequately account for regions consisting of intermediate degrees of MF resulting from partial volume effect as well as several other confounding factors including high contrast dosage, circulation kinetics, metabolic function, and surface coil proximity. While quantitative imaging techniques, such as T1-mapping and/or ECV mapping [29], could potentially improve contrast differentiation between MF and healthy myocardium, these pulse sequences are often limited in terms of spatial resolution, which would further accentuate the partial volume effect. Moreover, quantitative imaging sequences are typically optimized for imaging of the LV rather than the thin-walled LA. Such methods are also considerably slow to acquire, requiring several inversions applied between 6–8 heart beats to acquire anywhere from 8–11 raw images that are used to reconstruct a single two-dimensional map. Thus, full volume LA imaging using quantitative pulse sequences is not currently feasible.

The tachycardia experienced by canines also impacted the ability to acquire LGE CMR images in ventricular diastole, introducing substantial motion artifacts in post-pacing images and leading to challenges in the manual segmentation of the atrial chamber. These challenges were mitigated by registering the post-pacing images with uncorrupted post-mortem in situ images. Nevertheless, manual segmentation of the canine LA region is a complex task and requires anatomical expertise, which can result in significant variation in measurements computed using the IIR thresholding technique between different operators and among multiple attempts by a single operator. This is demonstrated in our intra-/inter- observer variability analysis of %MF. Paced animals show variation in %MF measurements computed at post-pacing time points by both observers, wherein the ICC of absolute %MF differences (D_abs_) ranges between 0.48 to 0.99 across both operators. However, the mean ± SD of %MF measurements computed by both operators nevertheless demonstrates an increasing trend from baseline to post-pacing among both paced animals. In the control dog, Operator 1 computes a mean decrease while Operator 2 demonstrates a mean increase in %MF from baseline to post-pacing (mean ± SD). Operator 2 computes a significant increase at the post-pacing time point for attempt 2 in control dog 3, however, this is likely an outlier as compared to metrics computed by the same operator in attempts 1 and 2. Nevertheless, this phenomenon demonstrates the impact that errors can have on %MF measurements.

Similarly, we demonstrate high and moderate spatial agreement of blood pool and LA myocardial wall regions, respectively, among both operators by DSC. However, poor spatial agreement is reported in MF volume segmentations provided by both operators, as demonstrated by the low DSC metrics reported in this region both operators in Table 5. This is likely due to the increasing necessity of operator interaction across the MF segmentation pipeline. The Boolean remove and Axial Dilate algorithms depend on the initial chamber segmentation but are imperfect in nature and require further alteration by the operator (i.e., removal of valves, septal regions, inclusion or exclusion of LA appendage, manual modifications to errors, etc.). Therefore, the decreasing trend of the DSC metric across blood pool, LA wall and MF regions is plausible, and in keeping with other studies [30]. Nevertheless, better spatial agreement is demonstrated by the boundary F1 score of the MF region, which indicates more consistency in the boundary-wise detection of MF (i.e., across the segmented edges, rather than the entire region). The BF score ranges between 29.77–87.09% across the MF volumes computed by both operators in our variability analysis, which is vastly superior to the DSC (entire region) metrics computed across both operators, which ranges between 0.28–34.26%. Moreover, moderate to high agreement was denoted by ICC and R metrics computed across both operators, which indicates that the percentages (i.e., not the spatial volumes) of MF computed by both operators demonstrate some consistency. However, the ICC and R metrics are computed using a single value (the percentage of MF) as opposed to a volume. Thus, spatial agreement of %MF is not taken into consideration in the computation of these metrics. Improved spatial agreement of MF volumes may be facilitated through the development of additional segmentation pipelines wherein minimal observer interaction is required.

There are several limitations in this study. Firstly, our sample size of nine experimental and three control dogs is small. However, we have expressed the results on a subject-by-subject basis to demonstrate an increasing trend across time points in experimental dogs, and an absence of this trend across control dogs. In this study, we consider LGE of the LA to be indicative of the presence of MF. However, late enhancement may also be used to identify other pathophysiological conditions that manifest in expansion of the myocardial space, such as amyloid or myocardial edema. Thus, enhanced regions of the LA are assumed to be MF while in theory, these are areas of expanded myocardial space that could be a product of another disease process. However, through histopathological analysis we confirmed the presence of LA myocardial MF. Therefore, classifying enhanced areas as MF was a reasonable assumption.

We also acknowledge the limitations involved in quantification of MF relating to the spatial resolution of LGE-CMR images. Although 3D LGE-CMR slice thickness and spatial resolution were <1mm as demonstrated in Table 1, the typical thickness of the left atrial wall generally ranges between 2–4 mm; thus, the LGE-CMR method bears the risk of wrongly characterizing adjacent tissue as myocardium which would further confound measurements of MF. Moreover, the reliance of operator precision in correctly segmenting the myocardial wall further elicits this risk. While respiratory and cardiac navigated pulse sequences improve spatial resolution to mitigate partial volume, these sequences are additionally limited by image artifacts and motion-induced respiratory and/or cardiac blurring. Therefore, MF volumes computed in this study may be confounded by image quality. While modifications and/or alternative pulse sequences could address these limitations, these options are often limited to scanners with optimized parameters for humans. The heart rates of canines are already considerably higher than typical human heart rates, which in itself limits the possible modifications to image acquisition protocols using cardiac gated pulse sequences, designed to acquire image data in the diastolic phase of the animal’s heart rate. Given the fact that the pulse sequence is gated to the animal’s unalterable heart rate, very few options for pulse sequence parameter modifications were available. Pulse sequences that mitigate the effects of partial volume, motion and blurring due to arrhythmic heart beats and suboptimal spatial resolution should be developed in further studies.

We also faced limitations in the correspondence of LA enhancement results derived from CMR images and histopathological quantification results. Inherent differences in image acquisition prevented us from doing more rigorous analysis of the relationship between the CMR results to the histopathological results. Because the %MF reported in histopathological imaging is reflective of a single slice area measurement, we computed slice-wise %MF from the slice containing the largest amount of MF in LGE-CMR in order to better compare the two modalities. However, we faced limitations in this comparison. Firstly, we were unable to compute slice-wise %MF measurements from the same slice(s) as measured in histopathology. This was because of inherent differences between image acquisition capabilities of each modality. Moreover, it was not feasible to match the slice orientation and location of ex-vivo histopathological images to in-vivo and in-situ LGE-CMR. Ideally, a registration of post-pacing and/or in-situ images to histopathological and/or ex-vivo images would allow direct spatial and geometric comparison of CMR and histological quantification. However, differences among these images are too numerous for this type of analysis to be attempted. For example, slice thickness of CMR images is 0.975 mm compared to 4 μm in histopathological images. That is, the slice thickness of CMR images is close to 1000 times larger than the slice thickness of histopathological images. Likewise, pixel-wise resolution of CMR images is on the order of millimetres as compared to micrometers in histopathological imaging, making direct quantitative comparison of fibrosis volumes problematic. Similarly, volumetric comparison of LA fibrosis measurements in histopathological imaging vs. CMR imaging was not possible in this study, as histopathological sampling of the entire LA volume was not feasible. Instead, a maximum of 7 slices were sampled for histopathological analysis, which may not be reflective of the measurement of fibrosis across the entire LA. Nevertheless, histopathological imaging confirmed the presence of fibrosis in the LA myocardium and therefore served as validation for MF measurements derived by CMR imaging using the IIR thresholding method. Lastly, histopathological imaging of each dog was not performed due to resource constraints, which limited our analysis of fibrosis trends across each sample group.

## Conclusion

Atrial fibrillation (AF) typically co-exists with myocardial fibrosis (MF); but underlying pathophysiological factors that contribute to AF are yet to be fully understood, making the definitive causal relationship controversial. In this study, we demonstrate that persistent AF leads to an increase in MF using quantification metrics based on a normalized image intensity ratio (IIR) in 3D IR-FLASH CMR imaging in a canine model and histopathological validation. Quantification of MF using LGE-CMR technique remains a challenging task limited by numerous factors, including inherent image acquisition parameters and observer variability. Improved MF computation may be facilitated by development of arrhythmia insensitive CMR imaging and/or techniques for automated segmentation of LA myocardial volumes. Additional studies are needed to further corroborate the causality of AF and MF.

## Supporting information

S1 Appendix Supplementary tables, figures, and equations. This file contains per-animal %MF and D_abs_ results expressed on a subject-by-subject basis across full volume (S1 Table 1) and single slice (S1 Table 2) images. Additionally, several equations are defined with which we express results for our inter- and intra- user variability analysis (S1 Tables 3–6). We also express volumetric %MF measurements across different users and compute ICC and R variability metrics in S1 Table 7.
(DOCX)

S1 File 3D IR-FLASH LGE-CMR images (.nrrd file format) at baseline and post-device insertion timepoints for Control Canine 1. Please note, in-situ images were not acquired in control animals.
(ZIP)

S2 File 3D IR-FLASH LGE-CMR images (.nrrd file format) at baseline and post-device insertion timepoints for Control Canine 2. Please note, in-situ images were not acquired in control animals.
(ZIP)

S3 File 3D IR-FLASH LGE-CMR images (.nrrd file format) at baseline and post-device insertion timepoints for Control Canine 3. Please note, in-situ images were not acquired in control animals.
(ZIP)

S4 File 3D IR-FLASH LGE-CMR images (.nrrd file format) at baseline, post-pacing, and in-situ (post-mortem) timepoints for Experimental Canine 1. (ZIP)

S5 File 3D IR-FLASH LGE-CMR images (.nrrd file format) at baseline, post-pacing, and in-situ (post-mortem) timepoints for Experimental Canine 2. (ZIP)

S6 File 3D IR-FLASH LGE-CMR images (.nrrd file format) at baseline, post-pacing, and in-situ (post-mortem) timepoints for Experimental Canine 3. (ZIP)

S7 File 3D IR-FLASH LGE-CMR images (.nrrd file format) at baseline, post-pacing, and in-situ (post-mortem) timepoints for Experimental Canine 4. (ZIP)

S8 File 3D IR-FLASH LGE-CMR images (.nrrd file format) at baseline, post-pacing, and in-situ (post-mortem) timepoints for Experimental Canine 5. (ZIP)

S9 File 3D IR-FLASH LGE-CMR images (.nrrd file format) at baseline, post-pacing, and in-situ (post-mortem) timepoints for Experimental Canine 6. (ZIP)

S10 File 3D IR-FLASH LGE-CMR images (.nrrd file format) at baseline, post-pacing, and in-situ (post-mortem) timepoints for Experimental Canine 7. (ZIP)

S11 File 3D IR-FLASH LGE-CMR images (.nrrd file format) at baseline, post-pacing, and in-situ (post-mortem) timepoints for Experimental Canine 8. (ZIP)

S12 File 3D IR-FLASH LGE-CMR images (.nrrd file format) at baseline, post-pacing, and in-situ (post-mortem) timepoints for Experimental Canine 9. (ZIP)

S13 File Histopathological image (.tif file format) of experimental dogs 4, 5 and 9 and control dog 1. Two histopathological images are provided for experimental dogs 4 and 5.
(ZIP)
