# Supplementary material for: Assessment of left atrial fibrosis progression in canines following rapid ventricular pacing using 3D late gadolinium enhanced CMR images
Source: PLoS One. 2022 Jul 8;17(7):e0269592. doi: 10.1371/journal.pone.0269592 (PMC9269919; doi:10.1371/journal.pone.0269592)
Supplement: S1 Appendix — This file contains per-animal %MF and Dabs results expressed on a subject-by-subject basis across full volume (S1 Table 1) and single slice (S1 Table 2) images. Additionally, several equations are defined with which we express results for our inter- and intra- user variability analysis (S1 Tables 3–6). We also express volumetric %MF measurements across different users and compute ICC and R variability metrics in S1 Table 7. (DOCX) [file pone.0269592.s001.docx]

**Appendix S1**

**S1.1 Per-animal fibrosis measurements**

Due to our small sample size of 9 experimental and 3 control dogs, we also expressed fibrosis quantification results on a subject-by-subject basis. Per-animal results are demonstrated in S1 Table 1.

**S1 Table 1. Per-animal volumetric fibrosis measurements computed on 3D IR-FLASH LGE CMR using the IIR thresholding method.**

| **Subject** | **Baseline %MF over total LA myocardial volume** | **Post-pacing %MF over total LA myocardial volume** | **Absolute %MF difference (D_abs_)** |
| --- | --- | --- | --- |
| **Paced dogs** | | | |
| Dog 1 | 2.71% | 3.59% | 0.87% |
| Dog 2 | 0.99% | 2.00% | 1.10% |
| Dog 3 | 0.11% | 1.93% | 1.82% |
| Dog 4 | 2.25% | 4.33% | 2.08% |
| Dog 5 | 2.05% | 1.40% | -0.65% |
| Dog 6 | 2.56% | 2.20% | -0.36% |
| Dog 7 | 0.12% | 2.96% | 2.84% |
| Dog 8 | 0.38% | 3.76% | 3.38% |
| Dog 9 | 0.29% | 3.08% | 2.79% |
| **Control dogs** | | | |
| Control 1 | 0.05% | 0.72% | 0.67% |
| Control 2 | 0.39% | 0.95% | 0.56% |
| Control 3 | 1.64% | 1.12% | -0.52% |

LA fibrosis volumes (%MF) computed using the IIR technique are expressed as a percentage of MF across the overall LA myocardial volume and are shown at baseline and post-pacing (i.e., post device-insertion for the control group) for each animal. In the third column, we express the absolute difference of volumetric %MF D_abs_ between baseline and post device-insertion time points.

**S1 Table 2. Per-animal slice-wise fibrosis measurements computed on 3D IR-FLASH LGE CMR using the IIR thresholding method.**

| **Subject** | **Baseline %MF over single LA myocardial slice area** | **Post-pacing %MF over single LA myocardial slice area** | **Absolute %MF difference (D_abs_)** |
| --- | --- | --- | --- |
| **Paced dogs** | | | |
| Dog 1 | 11.30% | 34.46% | 23.16% |
| Dog 2 | 7.16% | 11.22% | 4.06% |
| Dog 3 | 3.22% | 16.61% | 13.39 % |
| Dog 4 | 10.63% | 22.51% | 11.88 % |
| Dog 5 | 6.04% | 5.13% | -0.91% |
| Dog 6 | 6.58% | 3.34% | -3.24% |
| Dog 7 | 1.63% | 8.19% | 6.56% |
| Dog 8 | 1.64% | 19.66% | 18.02% |
| Dog 9 | 1.16% | 8.38% | 7.22% |
| **Control dogs** | | | |
| Control 1 | 9.24% | 9.98% | 0.74% |
| Control 2 | 9.98% | 9.28% | -0.70% |
| Control 3 | 10.46% | 11.52% | 1.06% |

Slice-wise (i.e., area-wise) LA fibrosis measurements are expressed as a percentage of MF (%MF) relative to the total myocardial area over the slice with which the greatest extent of fibrosis was shown. Area-wise %MF measurements are shown at baseline and post-pacing (i.e., post device-insertion for the control group) for each canine. In the third column, we express the absolute difference of area-wise %MF D_abs_ between baseline and post-pacing.

**S1.2. Variability analysis: computation of DSC, boundary F1 score, precision, and recall**

MF volumes were segmented as per our pipeline (see main text Figure 1) by two operators for the inter/intra- operator analysis in a sub-set of three animals (2 experimental, 1 control). The Dice Similarity Coefficient (DSC) and boundary F1 (BF) score were computed on segmentations of the myocardial blood pool chamber, myocardial LA wall and MF volumes across both operators, as demonstrated in Tables A and B, respectively. The $DSC$ is given as follows:

$DSC = \frac{2|A\cap B|}{|A| + |B|}$ (1)

where regions A and B are represented as two sets, |A∩B| is the intersection of A and B, and |A| represents the cardinality (i.e., the number of elements) in set A.

The BF score is given as follows:

$BF = 2\frac{Precision \times Recall}{Precision + Recall}$ (2)

where precision is a measure of the true positive instances among the amount of observed positive instances, while recall is a measure of the number of true positive instances compared to the actual number of positive instances. Given segmentation regions A and B, precision and recall are computed as follows:

$Precision = \frac{TP}{TP + FP}$ (3.1) $Recall = \frac{TP}{TP +FN}$ (3.2)

where a true positive (TP) instance is representative of a voxel that is found in both segmentation A and segmentation **B,** a false positive (FP) is a voxel that is included in segmentation B but not in segmentation A, and a false negative (FN) is indicative of a voxel that is not included in segmentation B but is included in segmentation A.

The precision, recall and BF metrics are computed based on object boundaries (i.e., edges) as opposed to the entire foreground regions. The BF score shows how much the boundary of segmentation B overlaps with the boundary of segmentation A. A distance tolerance of 2 voxels is used in the calculation of this measure, i.e., that the boundary of region B is considered a true positive if it is within two pixels of a boundary of region A.

The BF score is given as a percentage, wherein 0% BF indicates no overlap, and 100% BF indicates perfect overlap, respectively, between the boundaries of segmentations A and B. The BF score is not equivalent to the Dice Similarity Coefficient (DSC), which computes similarity based on the entire region, not just the edges. BF scores for blood pool, LA myocardial wall and MF are given in S1 Table 3. Precision and recall metrics are expressed in Tables 4 and 5, respectively.

The relative error index is a normalized measure of the difference in volume of the foreground of region A versus the foreground of region B. The relative error index RE is given as follows:

$RE = \frac{|V_{A} - V_{B}|}{V_{A}}$ (4)

where V_A_ is the volume of segmentation A and V_B_ is volume of segmentation B. RE is expressed as a percentage, where values close to 0% RE indicates that the two volumes are similar, and 100% RE indicates that the two volumes are considerably different. The RE metric computed on the blood pool chamber, LA myocardial wall and MF volumes are expressed in S1 Table 6.

**S1 Table 3. Average boundary F1 scores computed on three segmentation attempts by two operators.**

|  | | **Paced dog 1** | | **Paced dog 3** | | **Control dog 3** | |
| --- | --- | --- | --- | --- | --- | --- | --- |
| **Operator 1** | **Region** | **Baseline** | **Post-pacing** | **Baseline** | **Post-pacing** | **Baseline** | **Post-pacing** |
|  | BF Blood pool (%) | 97.37 ± 2.82 | 95.95 ± 2.46 | 99.88 ± 0.11 | 99.14 ± 0.35 | 99.65 ± 0.08 | 99.88 ± 0.15 |
|  | BF LA wall (%) | 97.46 ± 2.11 | 92.06 ± 3.03 | 98.82 ± 0.78 | 98.27 ± 0.85 | 98.92 ± 0.88 | 99.44 ± 0.42 |
|  | BF MF volume (%) | 53.57 ± 31.19 | 66.72 ± 23.70 | 22.97 ± 21.15 | 57.11 ±34.17 | 59.31 ± 25.13 | 48.77 ± 34.89 |
| **Operator 2** | BF Blood pool (%) | 98.91 ± 0.73 | 99.52 ± 0.51 | 99.37 ± 1.00 | 98.46 ± 1.21 | 99.96 ± 0.05 | 99.94 ± 0.06 |
|  | BF LA wall (%) | 98.78± 1.02 | 99.35 ± 0.18 | 99.67 ± 0.27 | 98.84 ± 1.01 | 99.86 ± 0.14 | 99.89 ± 0.08 |
|  | BF MF volume (%) | 79.81± 15.42 | 87.31±5.08 | 80.61 ± 3.34 | 59.41 ± 27.35 | 84.64±2.02 | 94.22±0.91 |
| **Operator 1 vs. Operator 2** | BF Blood pool (%) | 97.95 ± 1.22 | 96.74 ± 2.89 | 99.26 ± 0.65 | 96.77 ± 1.26 | 98.08 ± 0.41 | 99.33 ± 0.24 |
|  | BF LA wall (%) | 98.05 ± 0.92 | 95.30 ± 3.47 | 98.83 ± 0.59 | 97.06 ± 1.45 | 97.86 ± 1.09 | 99.29 ± 0.28 |
|  | BF MF volume (%) | 87.09 ± 15.15 | 79.36 ± 20.57 | 29.77 ± 12.22 | 30.59 ± 24.45 | 49.43 ± 16.84 | 59.39 ± 28.77 |

The BF score is expressed as a percentage (mean ± SD).

**S1 Table 4. Average precision computed on three segmentation attempts by two operators.**

|  | | **Paced dog 1** | | **Paced dog 3** | | **Control dog 3** | |
| --- | --- | --- | --- | --- | --- | --- | --- |
| **Operator 1** | **Region** | **Baseline** | **Post-pacing** | **Baseline** | **Post-pacing** | **Baseline** | **Post-pacing** |
|  | Precision Blood pool (%) | 99.44 ± 0.38 | 94.82 ± 3.40 | 99.93 ± 0.01 | 99.07 ± 0.43 | 100 ± 0.43 | 99.98 ± 0.03 |
|  | Precision LA wall (%) | 99.07 ± 0.82 | 90.22 ± 7.68 | 98.20 ± 1.59 | 97.89 ± 2.35 | 97.95 ± 1.72 | 99.83 ± 0.28 |
|  | Precision MF volume (%) | 70.40 ± 28.10 | 60.81 ± 32.76 | 61.07 ± 27.43 | 97.70 ± 2.85 | 95.08 ± 5.82 | 94.51 ± 9.50 |
| **Operator 2** | Precision Blood pool (%) | 99.78 ± 0.06 | 99.39 ± 0.70 | 100 ± 0 | 98.11 ± 1.69 | 99.92 ± 0.09 | 99.97 ± 0.04 |
|  | Precision LA wall (%) | 97.87 ± 1.87 | 99.17 ± 0.18 | 99.97 ± 0.03 | 98.69 ± 1.13 | 99.92 ± 0.07 | 99.97 ± 0.03 |
|  | Precision MF volume (%) | 72.91 ± 22.87 | 82.10 ± 8.27 | 74.96 ± 0.82 | 53.88 ± 27.67 | 88.49 ± 4.38 | 92.88 ± 3.78 |
| **Operator 1 vs. Operator 2** | Precision Blood pool (%) | 99.26 ± 0.37 | 95.78 ± 3.55 | 99.72 ± 0.40 | 97.83 ± 2.16 | 95.99 ± 0.76 | 99.64 ± 0.47 |
|  | Precision LA wall (%) | 99.00 ± 0.91 | 93.51 ± 5.21 | 98.76 ± 1.13 | 95.92 ± 2.35 | 97.16 ± 1.67 | 99.63 ± 0.48 |
|  | Precision MF volume (%) | 92.33 ± 10.50 | 73.36 ± 24.00 | 60.63 ± 28.43 | 59.47 ± 34.99 | 69.73 ± 27.69 | 81.40 ± 13.86 |

Precision is expressed as a percentage (mean ± SD).

**S1 Table 5. Average recall computed on three segmentation attempts by two operators.**

|  | | **Paced dog 1** | | **Paced dog 3** | | **Control dog 3** | |
| --- | --- | --- | --- | --- | --- | --- | --- |
| **Operator 1** | **Region** | **Baseline** | **Post-pacing** | **Baseline** | **Post-pacing** | **Baseline** | **Post-pacing** |
|  | Recall Blood pool (%) | 95.40 ± 5.29 | 97.13 ± 1.59 | 99.83 ± 0.20 | 99.21 ± 0.34 | 99.74 ± 0.27 | 99.79 ± 0.32 |
|  | Recall LA wall (%) | 95.92 ± 3.34 | 94.32 ± 2.14 | 99.45 ± 0.37 | 98.70 ± 1.52 | 99.93 ± 0.01 | 99.06 ± 0.58 |
|  | Recall MF volume (%) | 47.60 ± 41.09 | 79.19 ± 9.76 | 18.94 ± 22.57 | 48.02 ± 42.63 | 48.66 ± 33.74 | 42.26 ± 42.99 |
| **Operator 2** | Recall Blood pool (%) | 98.06 ± 1.45 | 99.65 ± 0.33 | 98.75 ± 1.97 | 98.83 ± 1.17 | 100 ± 0.01 | 99.92 ± 0.08 |
|  | Recall LA wall (%) | 99.73 ± 0.28 | 99.53 ± 0.18 | 99.37 ± 0.52 | 98.99 ± 1.14 | 99.80 ± 0.20 | 99.81 ± 0.15 |
|  | Recall MF volume (%) | 90.42 ± 7.11 | 93.59 ± 4.12 | 87.49 ± 8.45 | 67.15 ± 25.23 | 81.62 ± 7.60 | 95.83 ± 4.47 |
| **Operator 1 vs. Operator 2** | Recall Blood pool (%) | 96.69 ± 2.20 | 97.73 ± 2.22 | 98.81 ± 1.32 | 95.84 ± 3.25 | 96.69 ± 2.20 | 97.73 ± 2.22 |
|  | Recall LA wall (%) | 97.12 ± 1.51 | 93.51 ± 1.94 | 98.92 ± 0.65 | 98.26 ± 1.30 | 97.12 ± 1.51 | 97.23 ± 1.94 |
|  | Recall MF volume (%) | 83.49 ± 19.47 | 88.70 ± 12.82 | 21.72 ± 10.62 | 21.79 ± 19.22 | 83.49 ± 19.47 | 88.70 ± 12.82 |

Recall is expressed as a percentage (mean ± SD).

**S1 Table 6. Average relative error (RE) index computed on blood pool, LA wall and MF volumes across different operators.**

|  | | **Paced dog 1** | | **Paced dog 3** | | **Control dog 3** | |
| --- | --- | --- | --- | --- | --- | --- | --- |
| **Operator 1** | **Region** | **Baseline** | **Post-pacing** | **Baseline** | **Post-pacing** | **Baseline** | **Post-pacing** |
|  | RE Blood pool (%) | 29.06 ± 22.82 | 19.05 ± 12.36 | 6.00 ± 2.59 | 4.41 ± 2.46 | 14.84 ± 11.86 | 3.13 ± 1.58 |
|  | RE LA wall (%) | 23.02 ± 17.33 | 19.68 ± 12.89 | 10.99 ± 4.56 | 12.78 ± 5.50 | 18.98 ± 9.73 | 3.15 ± 2.19 |
|  | RE MF volume (%) | 18.87 ± 7.82 | 33.57 ± 16.64 | 81.40 ± 31.80 | 72.69 ± 42.73 | 47.25 ± 48.37 | 32.12 ± 54.57 |
| **Operator 2** | RE Blood pool (%) | 5.03 ± 3.32 | 16.14 ± 7.11 | 4.67 ± 1.97 | 7.20 ± 3.35 | 3.89 ± 2.63 | 4.60 ± 3.66 |
|  | RE LA wall (%) | 9.28 ± 3.98 | 3.27 ± 1.78 | 16.00 ± 6.91 | 3.62 ± 1.82 | 4.06 ± 1.75 | 8.11 ± 5.57 |
|  | RE MF volume (%) | 21.94 ± 8.80 | 13.62 ± 7.15 | 31.52 ± 12.17 | 42.82 ± 13.82 | 5.73 ± 2.92 | 31.03 ± 14.29 |
| **Operator 1 vs. Operator 2** | RE Blood pool (%) | 20.86 ± 21.70 | 15.75 ± 9.72 | 10.84 ± 4.56 | 5.60 ± 3.41 | 12.89 ± 4.93 | 20.74 ± 3.25 |
|  | RE LA wall (%) | 17.82 ± 14.32 | 14.00 ± 10.78 | 28.54 ± 9.79 | 29.68 ± 6.51 | 20.37 ± 12.04 | 18.61± 5.17 |
|  | RE MF volume (%) | 23.43 ± 13.22 | 25.40 ± 16.38 | 89.49 ± 8.26 | 72.80 ± 23.76 | 63.57 ± 29.13 | 47.57 ± 43.24 |

RE metrics were computed on three segmentation attempts performed by two operators at each time point in a subsample of three animals. The RE is expressed as a percentage (mean ± SD).

**S1.3. Observer variability: volumetric comparison of %MF measurements across manual segmentation attempts**

MF volumes were segmented as per our pipeline (see main text Figure 1) by two operators for the inter/intra- operator analysis in a sub-set of three animals (2 experimental, 1 control). Fibrosis measurements were computed as a percentage of the overall LA myocardial volume at baseline and post-pacing (i.e., post device-insertion for the control group in order to compute the intraclass correlation coefficient (ICC) and inter-rater reliability (R). MF measurements at baseline, post-pacing, the absolute difference (D_abs_) and the ICC and R metrics are expressed across two operators in Table 7.

|  | | **Paced dog 1** | | | **Paced dog 3** | | | **Control dog 3** | | |
| --- | --- | --- | --- | --- | --- | --- | --- | --- | --- | --- |
|  |  | **Baseline** | **Post-pacing** | **Absolute difference (D_abs_)** | **Baseline** | **Post-pacing** | **Absolute difference (D_abs_)** | **Baseline** | **Post-pacing** | **Absolute difference (D_abs_)** |
| **Operator 1** | Attempt 1 MF% | 2.45 | 4.32 | 1.87 | 0.40 | 1.86 | 1.46 | 1.71 | 0.94 | -0.77 |
|  | Attempt 2 MF% | 2.23 | 4.84 | 2.61 | 1.35 | 2.75 | 1.40 | 1.54 | 1.31 | -0.23 |
|  | Attempt 3 MF% | 2.70 | 3.58 | 0.88 | 1.07 | 1.93 | 0.86 | 1.63 | 1.11 | -0.52 |
|  | Mean %MF±SD | 2.46 ± 0.23 | 4.25 ± 0.63 | 1.79 ± 0.89 | 0.94 ± 0.49 | 2.18 ± 0.49 | 1.24 ± 0.33 | 1.62 ± 0.09 | 1.12 ± 0.19 | -0.51 ± 0.27 |
|  | Median %MF±IQR | 2.45 ± 0.35 | 4.32 ± 0.94 | 1.87 ± 1.30 | 1.07 ± 0.71 | 1.93 ± 0.67 | 1.40 ± 0.45 | 1.63 ± 0.13 | 1.11 ± 0.28 | -0.52 ± 0.41 |
|  | ICC | 0.90 | 0.53 | 0.64 | 0.58 | 0.45 | 0.48 | 0.84 | 0.91 | 0.88 |
| **Operator 2** | Attempt 1 MF% | 2.24 | 3.11 | 0.87 | 2.00 | 2.78 | 0.78 | 1.34 | 1.28 | -0.06 |
|  | Attempt 2 MF% | 3.80 | 3.18 | -0.62 | 1.19 | 2.00 | 0.81 | 1.23 | 2.55 | 1.32 |
|  | Attempt 3 MF% | 3.82 | 3.93 | 0.11 | 1.89 | 2.70 | 0.81 | 1.30 | 1.49 | 0.19 |
|  | Mean %MF±SD | 3.29 ± 0.90 | 3.41 ± 0.45 | 0.12 ± 0.75 | 1.69 ± 0.43 | 2.49 ± 0.43 | 0.80 ± 0.02 | 1.29 ± 0.06 | 1.77 ± 0.68 | 0.48 ± 0.74 |
|  | Median %MF±IQR | 3.80 ± 1.18 | 3.18 ± 0.62 | 0.11 ± 1.11 | 1.89 ± 0.61 | 2.70 ± 0.59 | 0.81 ± 0.02 | 1.30 ± 0.08 | 1.49 ± 0.95 | 0.19 ± 1.03 |
|  | ICC | 0.40 | 0.68 | 0.71 | 0.64 | 0.52 | 0.99 | 0.93 | 0.41 | 0.49 |
| **R (Operator 1 vs. Operator 2**) | | 0.58 | 0.69 | 0.81 | 0.74 | 0.62 | 0.82 | 0.92 | 0.59 | 0.66 |

**S1 Table 7. Fibrosis measurements computed on a subset of animals three times by two operators.**

Two operators (redacted) computed the volumetric %MF at baseline and post-pacing time points across for three segmentation attempts for a subsample of 2 experimental and 1 control dog. The %MF is expressed per each segmentation attempt and the three-way mean and median measurements are demonstrated per each operator. We also computed the intraclass correlation coefficient (ICC) and inter-rater reliability (R) metrics using the %MF (i.e., not the MF volume) among the three attempts per operator.
