# Supplementary figures and images for: Assessment of left atrial fibrosis progression in canines following rapid ventricular pacing using 3D late gadolinium enhanced CMR images
Source: PLoS One. 2022 Jul 8;17(7):e0269592. doi: 10.1371/journal.pone.0269592 (PMC9269919; doi:10.1371/journal.pone.0269592)

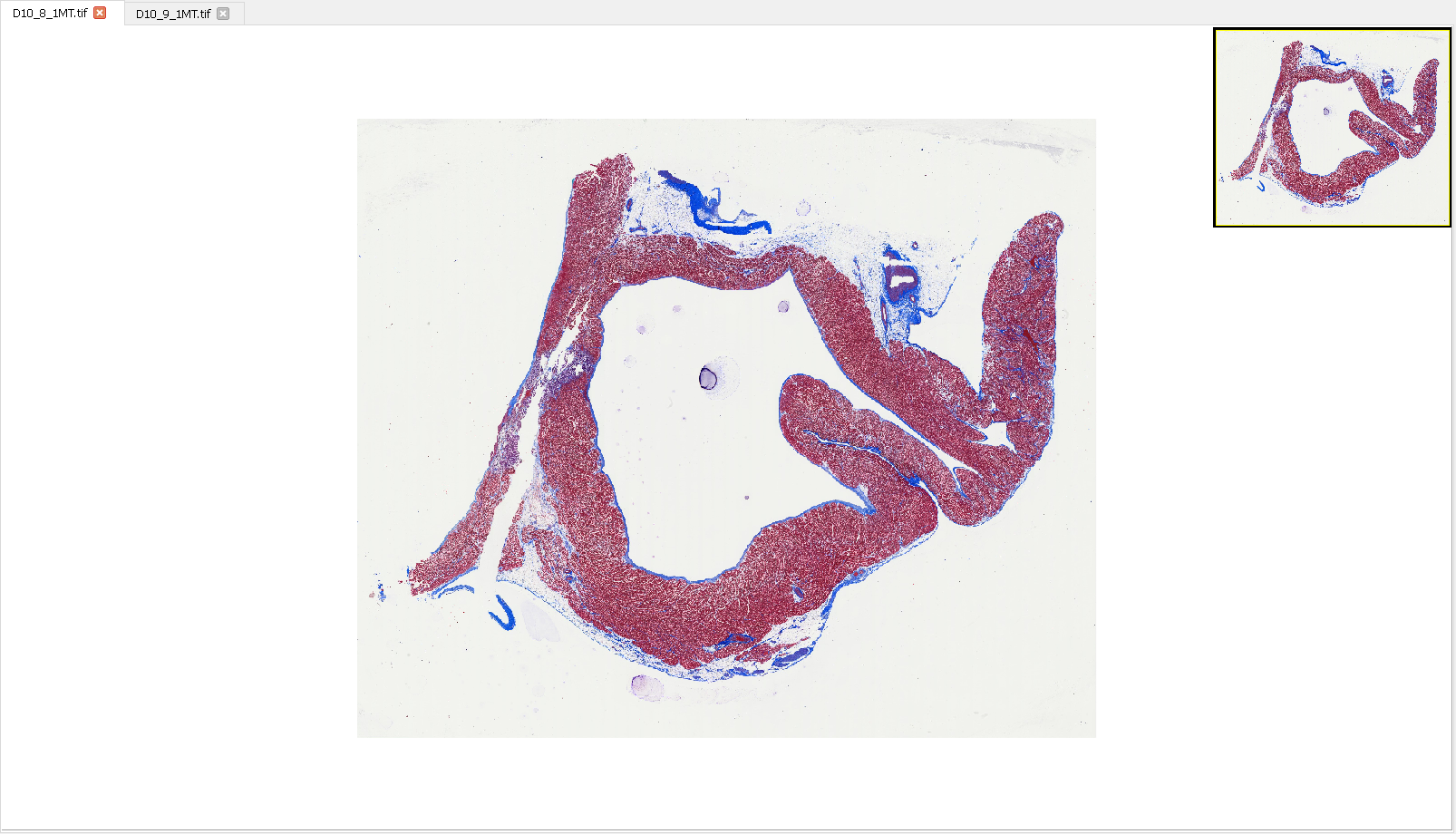

Supplement: S13 File — Two histopathological images are provided for experimental dogs 4 and 5. (ZIP) [file pone.0269592.s014.zip › HISTO/Control_D10_8_1MT_screenshot.tif]

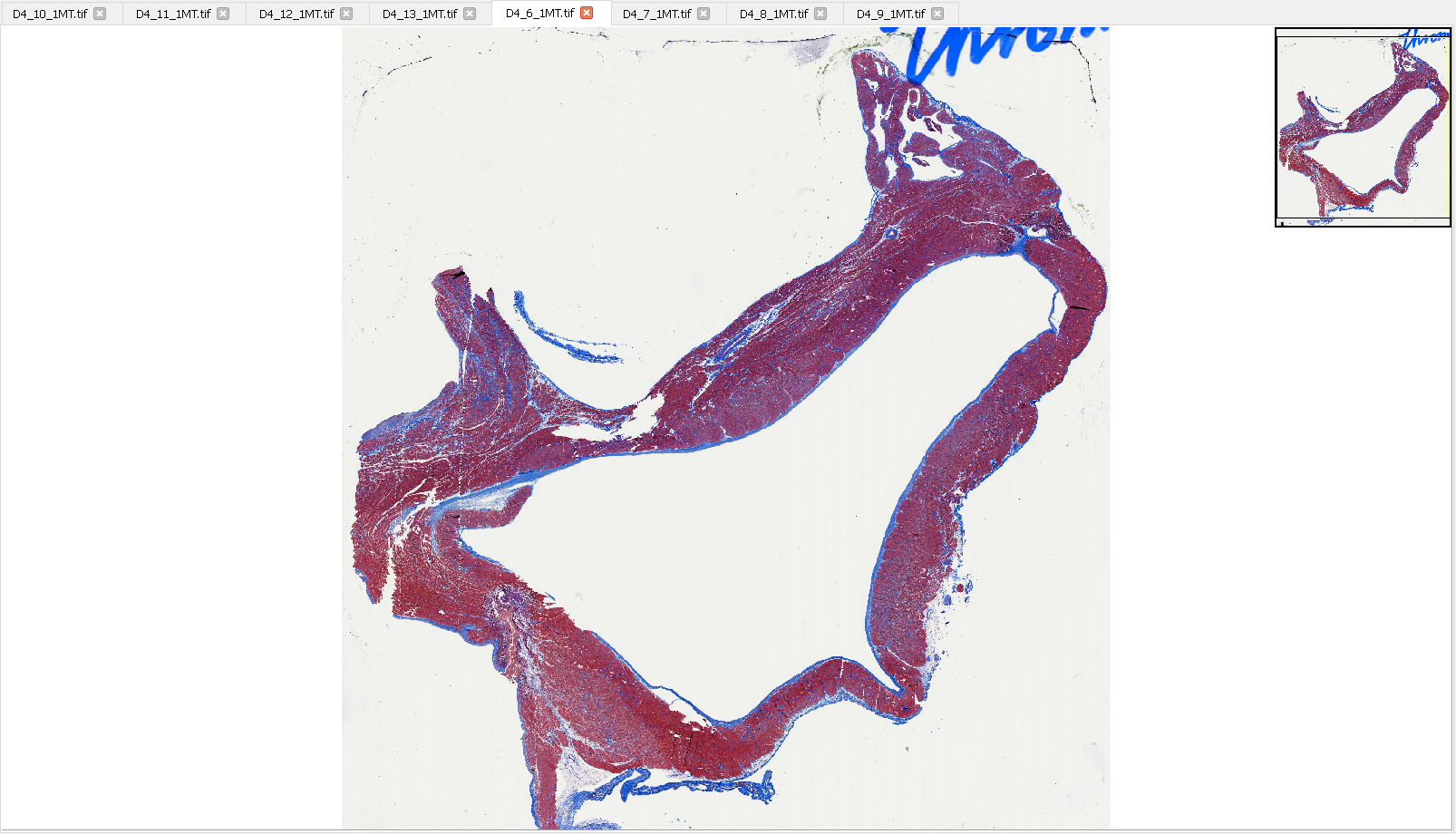

Supplement: S13 File — Two histopathological images are provided for experimental dogs 4 and 5. (ZIP) [file pone.0269592.s014.zip › HISTO/Exp_D4_6_1MT_screenshot.tif]

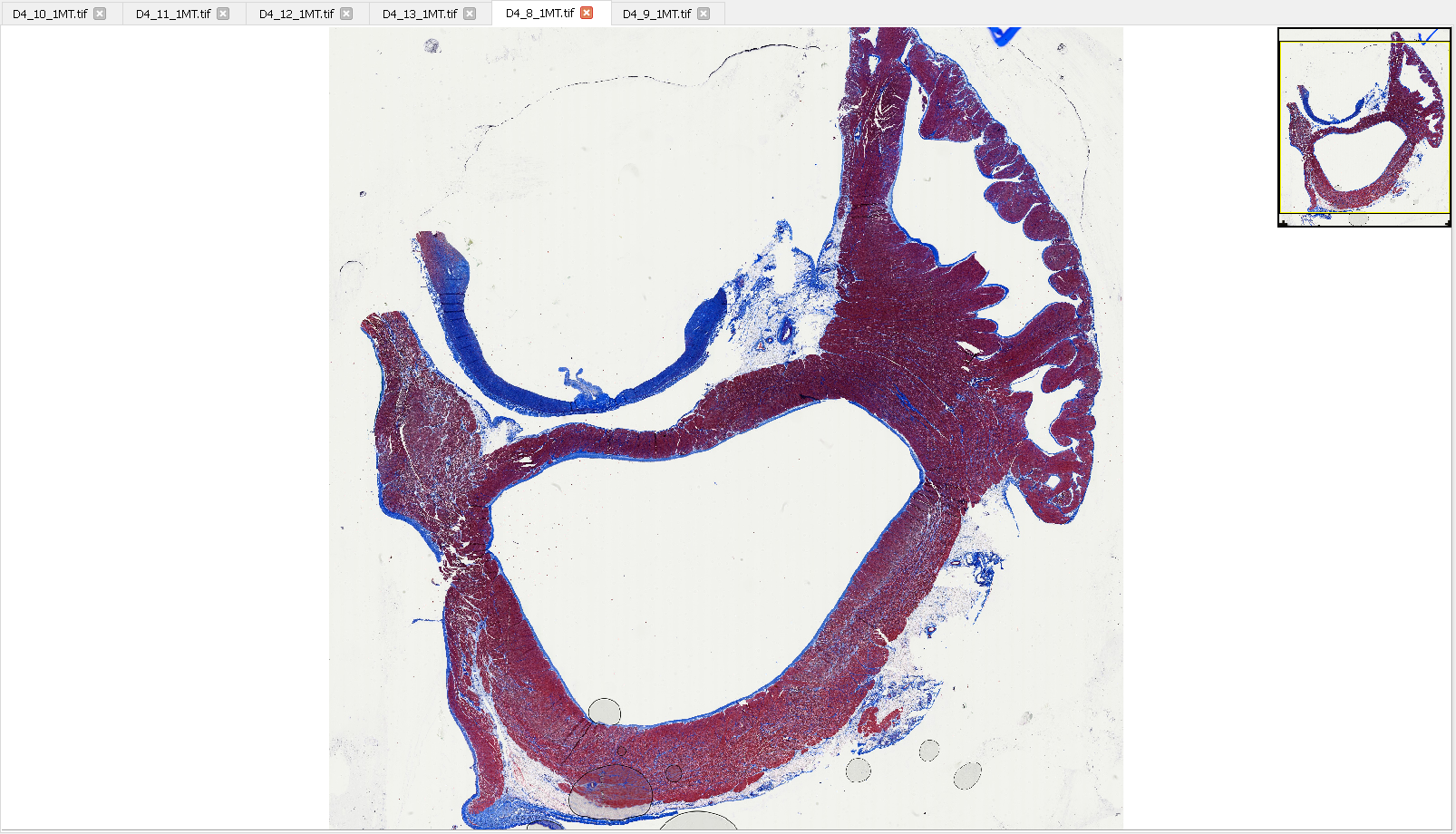

Supplement: S13 File — Two histopathological images are provided for experimental dogs 4 and 5. (ZIP) [file pone.0269592.s014.zip › HISTO/Exp_D4_8_1MT_screenshot.tif]

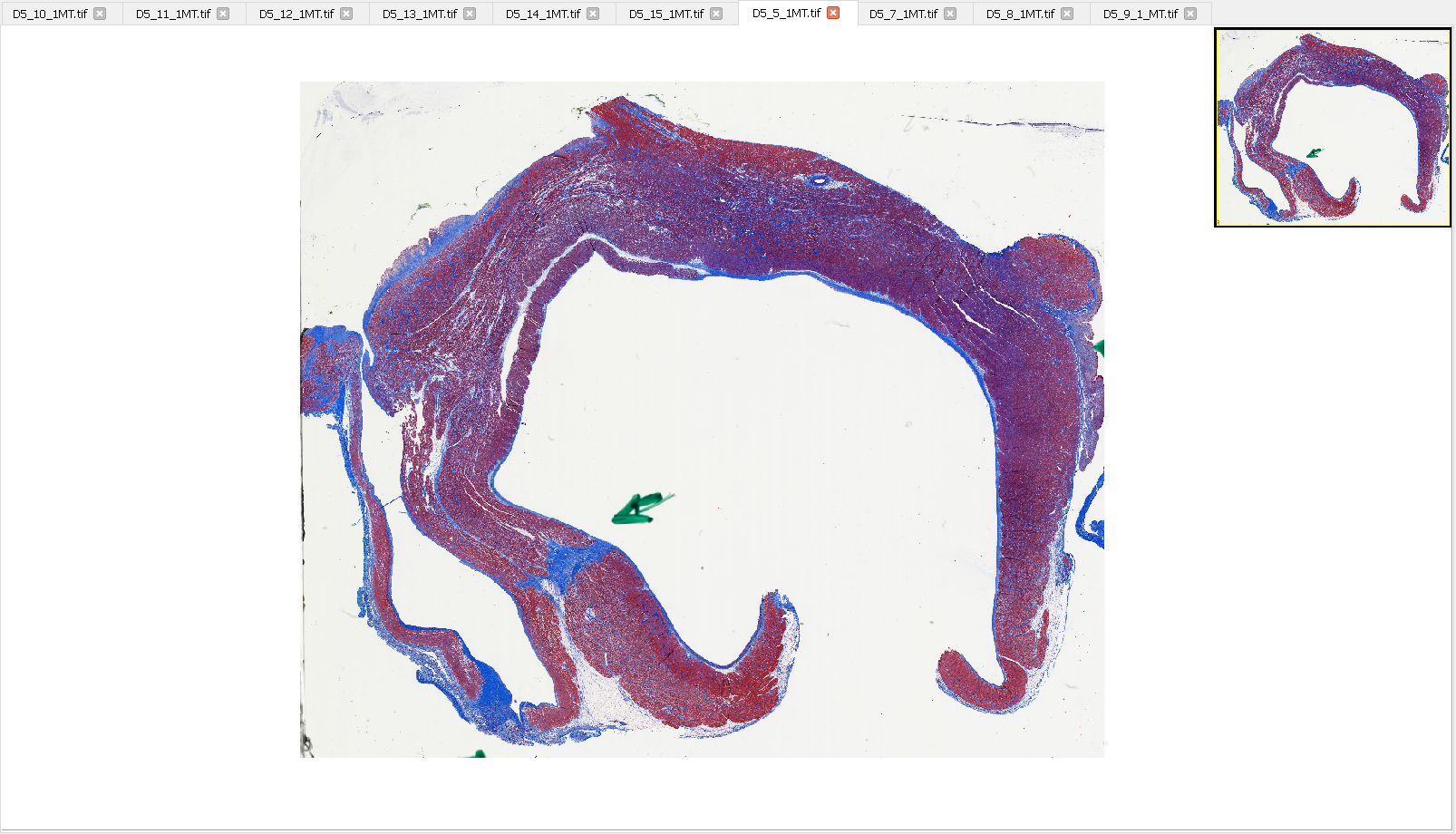

Supplement: S13 File — Two histopathological images are provided for experimental dogs 4 and 5. (ZIP) [file pone.0269592.s014.zip › HISTO/Exp_D5_5_1MT_screenshot.tif]

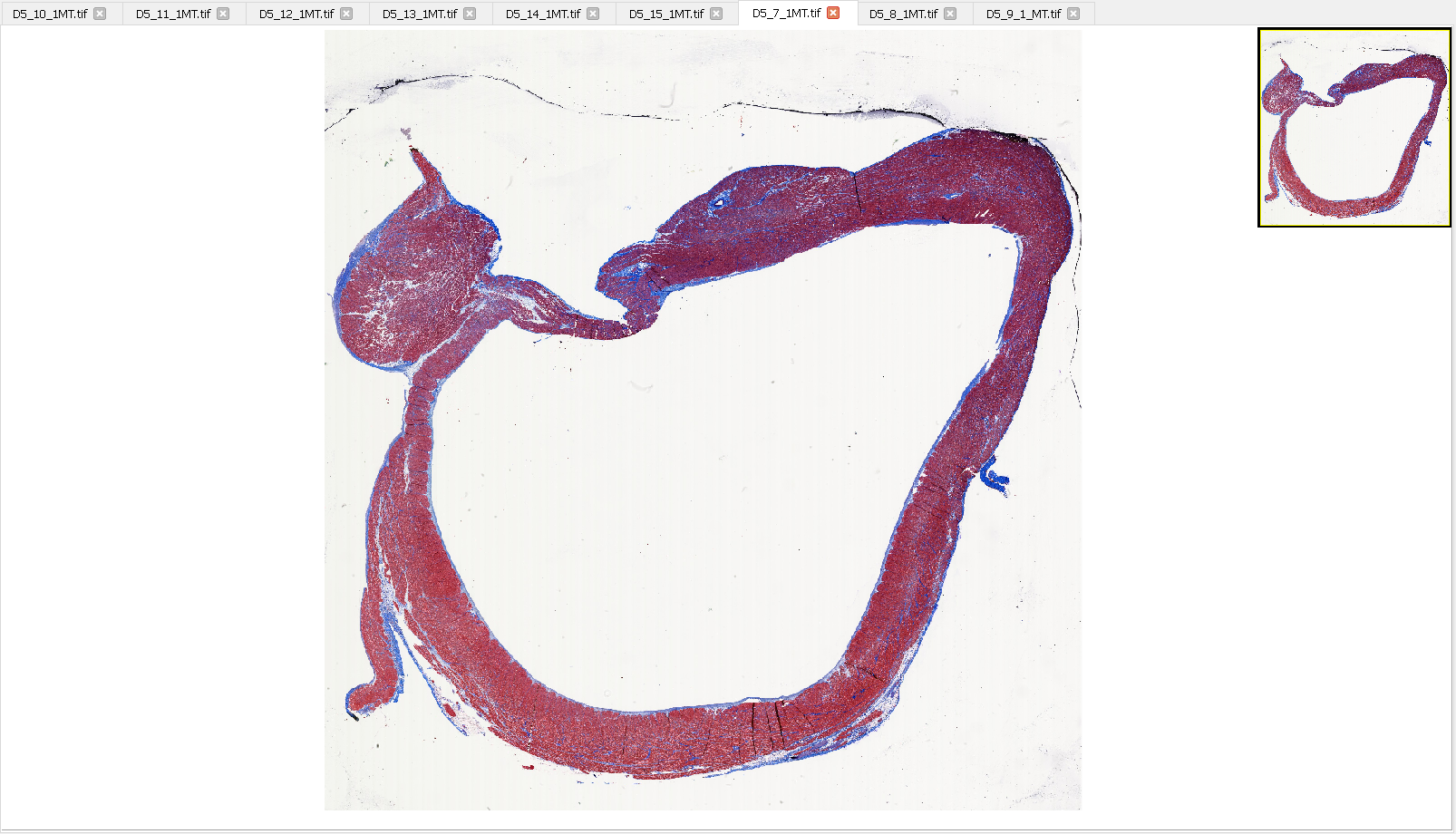

Supplement: S13 File — Two histopathological images are provided for experimental dogs 4 and 5. (ZIP) [file pone.0269592.s014.zip › HISTO/Exp_D5_7_1MT_screenshot.tif]

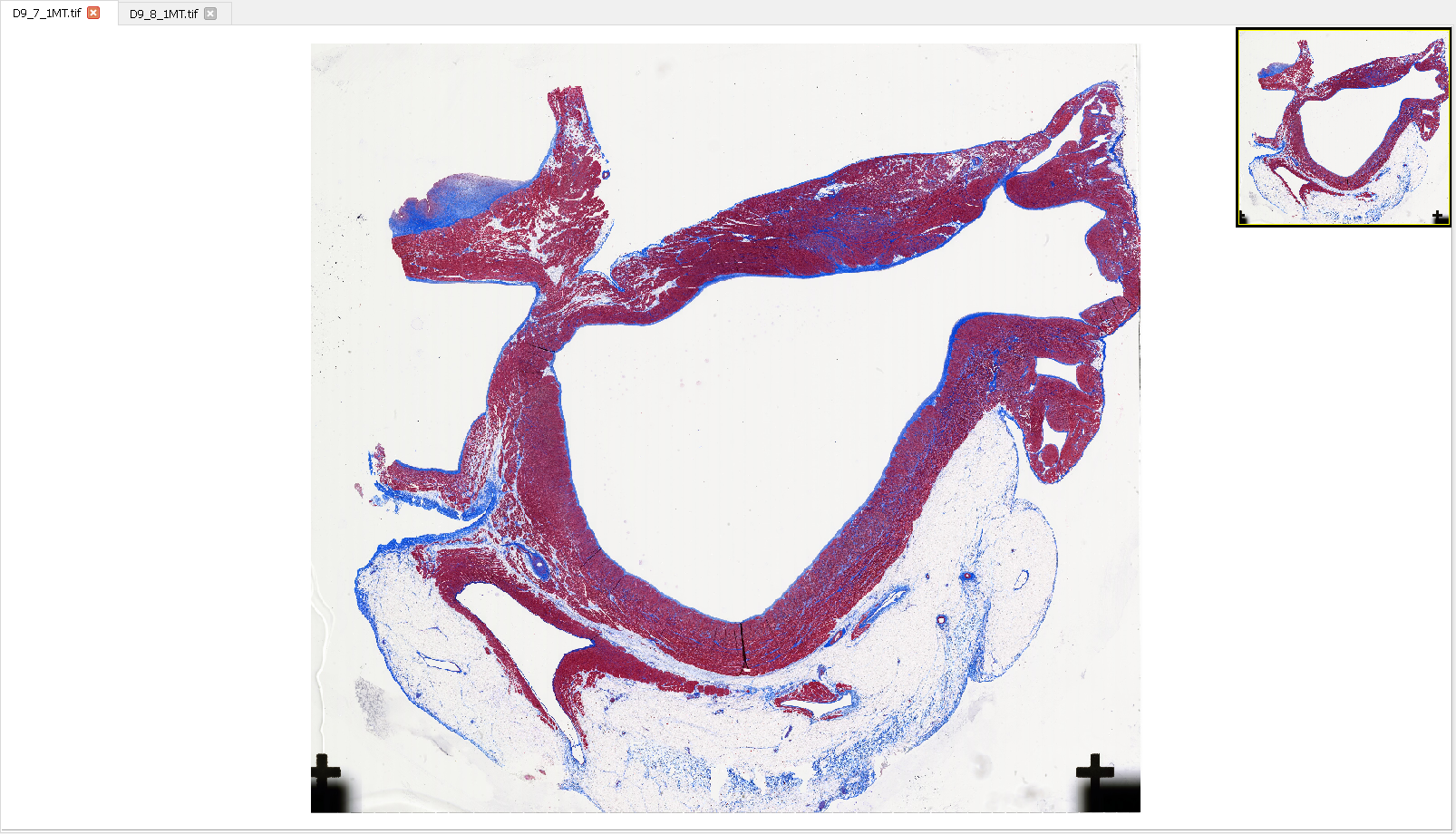

Supplement: S13 File — Two histopathological images are provided for experimental dogs 4 and 5. (ZIP) [file pone.0269592.s014.zip › HISTO/Exp_D9_7_1MT_screenshot.tif]
